# Differential impacts of ribosomal protein haploinsufficiency on mitochondrial function

**DOI:** 10.1083/jcb.202404084

**Published:** 2025-01-09

**Authors:** Agustian Surya, Blythe Marie Bolton, Reed Rothe, Raquel Mejia-Trujillo, Amanda Leonita, Qiuxia Zhao, Alia Arya, Yue Liu, Rekha Rangan, Yasash Gorusu, Pamela Nguyen, Can Cenik, Elif Sarinay Cenik

**Affiliations:** 1 Department of Molecular Biosciences, University of Texas at Austin, Austin, TX, USA

## Abstract

The interplay between ribosomal protein (RP) composition and mitochondrial function is essential for energy homeostasis. Balanced RP production optimizes protein synthesis while minimizing energy costs, but its impact on mitochondrial functionality remains unclear. Here, we investigated haploinsufficiency for RP genes (*rps-10*, *rpl-5*, *rpl-33*, and *rps-23*) in *Caenorhabditis elegans* and corresponding reductions in human lymphoblast cells. Significant mitochondrial morphological differences, upregulation of glutathione transferases, and SKN-1–dependent oxidative stress resistance were observed across mutants. Loss of a single *rps-10* copy reduced mitochondrial activity, energy levels, and oxygen consumption, mirrored by similar reductions in mitochondrial activity and energy levels in lymphoblast cells with 50% lower RPS10 transcripts. Both systems exhibited altered translation efficiency (TE) of mitochondrial electron transport chain components, suggesting a conserved mechanism to adjust mitochondrial protein synthesis under ribosomal stress. Finally, mitochondrial membrane and cytosolic RPs showed significant RNA and TE covariation in lymphoblastoid cells, highlighting the interplay between protein synthesis machinery and mitochondrial energy production.

## Teaser

Haploinsufficiency of DBA causative ribosomal protein genes caused significant mitochondrial morphological changes; only loss of rps-10 led to mitochondrial dysfunction—including reduced oxygen consumption, energy levels, and mitochondrial activity. These findings suggest a conserved mechanism linking ribosomal stress to mitochondrial function via altered mitochondrial translation.

## Introduction

The coordinated expression of ∼79 ribosomal proteins (RPs) is essential for cellular health and development. In humans, the haploinsufficiency or point mutations of RP genes leads to a range of ribosomopathies (Paolini et al., 2017), including Diamond-Blackfan anemia (DBA) (Ulirsch et al., 2018), and has been linked to an increased susceptibility to certain cancers, such as myelodysplastic syndromes and acute myeloid leukemia (Vlachos et al., 2012; Goudarzi and Lindström, 2016). With its hallmark features of hematological dysfunction and an increased risk of malignancies, DBA exemplifies the systemic consequences of RP deficits (Lipton, 2006; Ellis and Lipton, 2008).

DBA and other ribosomopathies are rare genetic disorders. However, hemizygous losses of RP genes are frequently observed (∼40%) in tumors (Ferreira et al., 2014; Ajore et al., 2017) and impact cellular proliferation and oncogenesis (Amsterdam et al., 2004; Kulkarni et al., 2017; Guimaraes and Zavolan, 2016). Specifically, RP mutations are associated with higher mutational load in T-cell acute lymphoblastic leukemia (T-ALL) patients (Sulima et al., 2019; Girardi et al., 2017). Hemizygous deletion of *RPL5* occurs in 11–34% of multiple tumor types, and reduced expression of this gene is correlated with poor survival in glioblastoma and breast cancer (Fancello et al., 2017; De Keersmaecker et al., 2013). Conversely, overexpression of RPL15 and RPL28 leads to increased metastatic growth (Ebright et al., 2020; Labriet et al., 2019). The phenotypes associated with these genetic disruptions allude to the roles that these proteins play beyond protein synthesis.

Considering the significant energy demands of ribosome biogenesis (Warner, 1990), mitochondrial function and ribosome production are interconnected to ensure optimal cellular energy equilibrium. A reciprocal connection between ribosomal and mitochondrial DNA copy number is observed across individuals (Gibbons et al., 2014). Moreover, mitochondrial dysfunction leads to retrograde signaling that alters the accumulation of extra chromosomal ribosomal DNA circles (Borghouts et al., 2004). One potential mechanistic link between these processes is RNAse MRP, which is involved both in the processing of ribosomal RNA in the nucleolus, and in priming DNA replication in mitochondria (Yuan et al., 1989; Topper and Clayton, 1990; Topper et al., 1992; Stohl and Clayton, 1992; Lee et al., 1996).

Other observations supporting the connection between ribosome biogenesis and mitochondrial function include: (1) translation of mitochondrial transcripts are reduced and mitochondrial structure and oxygen consumption are altered in response to the deletion of ribosome biogenesis factor, *Bud23*, in mouse cardiomyocytes (Baxter et al., 2020). (2) Yeast *Asc1* (*RACK1* ortholog) mutants, exhibit reduced translation of cytosolic and mitochondrial ribosome transcripts and lower fitness in a non-fermentable carbon source suggesting decreased mitochondrial activity (Thompson et al., 2016). (3) The inhibition of ribosomal RNA synthesis through the depletion of the RNA polymerase I component, RPOA-2, in *C. elegans* results in a significant decrease in mitochondrial RPs without affecting their transcript levels (Zhao et al., 2023; Freeman et al., 2023). These observations suggest that altering ribosome biogenesis could alter mitochondrial components or function across different species. Reciprocal to the evidence provided, the biogenesis of cytosolic ribosomes also requires functional mitochondria. For instance, Rli1p, a protein carrying Fe/S clusters and thus requiring mitochondrial protein machinery, is associated with ribosomes and Hcr1p, which is involved in 20S pre-rRNA processing and translation initiation (Kispal et al., 2005).

Interestingly, a notable parallel has been observed between DBA and Pearson syndrome, which results from mitochondrial DNA losses (Fontenay et al., 2006). Their symptoms are strikingly similar; in one instance, ∼5% of patients initially diagnosed with DBA were found to have significant mitochondrial DNA loss, leading to their reclassification as Pearson syndrome patients (Gagne et al., 2014). Similarly, expression analysis within a large family carrying a single-copy SNP variant in RPL11 (Narla et al., 2016; Carlston et al., 2017) suggested altered mitochondrial expression, indicating that coordination between mitochondria and ribosomes may be disrupted upon single-copy loss of RP genes (Panici et al., 2021).

Despite the known links between ribosome biogenesis and mitochondria, the ways in which mitochondrial function and oxidative stress relate to RP haploinsufficiency have yet to be explored. Mitochondria not only produce ATP but also play an important role in regulating oxidative stress through reactive oxygen (ROS) production. Disruptions in mitochondrial function can lead to oxidative stress (Jones, 2006).

In *C*.* elegans*, the transcription factor SKN-1 (homologous to mammalian *NRF2*) regulates the oxidative stress response by activating detoxification genes such as gluthatione S-transferases (*gst*) (Settivari et al., 2013; Blackwell et al., 2015). Changes in RP level may affect SKN-1 activity and oxidative stress pathways, but this connection hasn’t been thoroughly investigated. Previous studies hint at a connection between ribosome biogenesis and mitochondrial function in maintaining cellular homeostasis. However, the specific effects of RP haploinsufficiency on mitochondrial morphology and oxidative stress were not well characterized.

Here, we investigate the effects of single-copy loss for four RP genes (*rps-10*, *rpl-5*, *rpl-33*, and *rps-23*) in *C*.* elegans*, along with corresponding reductions in human lymphoblast cells. Our investigations revealed significant mitochondrial morphological alterations with increased oxidative stress resistance across these RP haploinsufficient mutants and a conserved mechanism that coordinates the translation of mitochondrial components in response to compromised ribosomal machinery. Notably, a reduction in the cytoplasmically assembled RPS-10 in *C. elegans* (*rps-10(0)/+* mutant) exhibited altered mitochondrial function and reduced cellular energy—a phenomenon mirrored by a 50% reduction in RPS10 abundance in human cells. These observations are further supported by significant expression covariation between mitochondrial membrane components and RPs across lymphoblastoid cells derived from a diverse group of individuals, suggesting an adaptive conserved mechanism of mitochondrial function in response to ribosomal expression alterations.

## Results

### Developmental and physiological consequences of ribosomal protein gene haploinsufficiency in *C*.* elegans*

**Figure 1.**
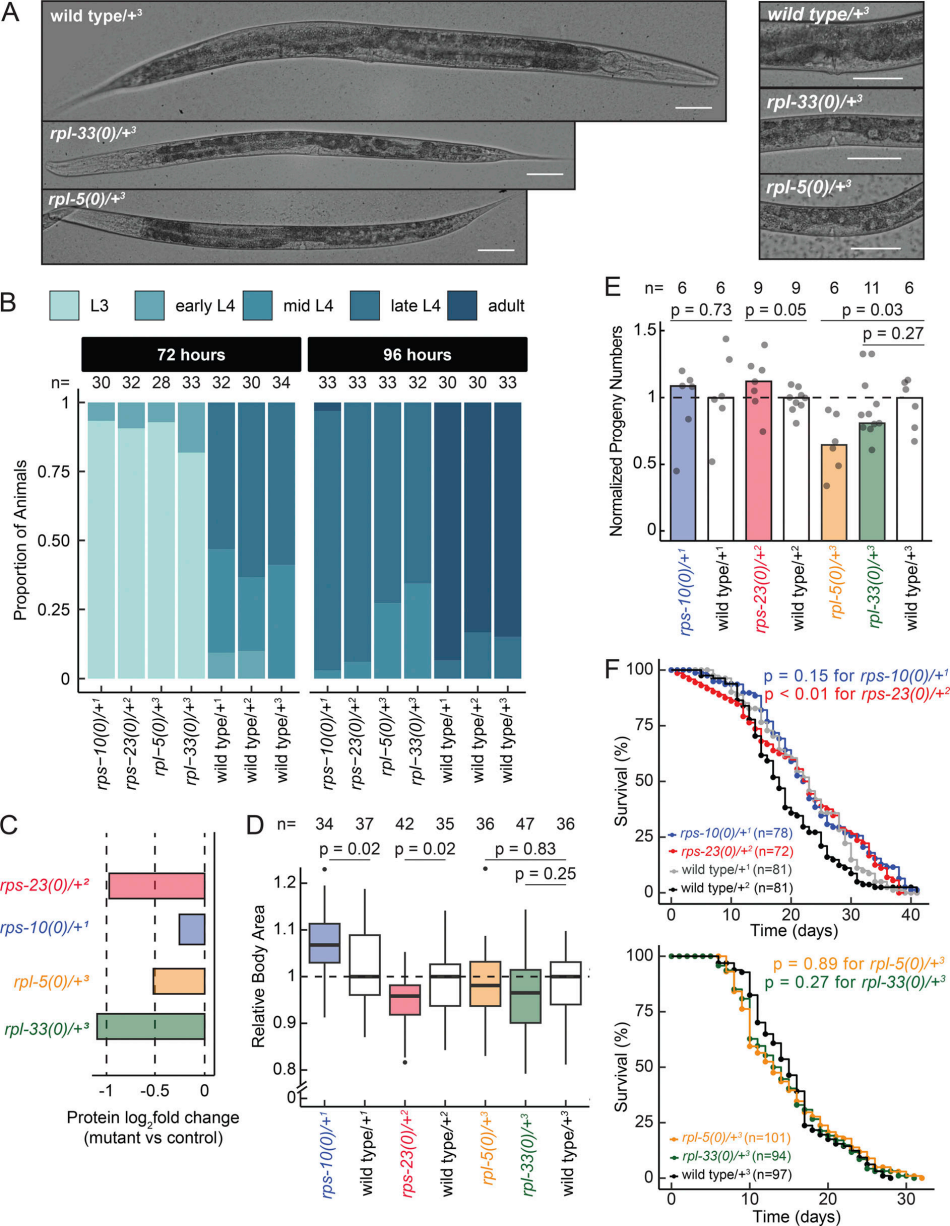
**Ribosomal protein haploinsufficiency results in developmental delays and variable body size without affecting lifespan in *C. elegans*. (A)** The development of single copy large subunit ribosomal protein (RP) mutants alongside their wild-type counterparts after 96 h of incubation from embryo at 16°C. Images show ∼1 day of growth delay (left) and differences in vulval development (right), with a scale bar of 50 µm. Differential interference contrast images were taken with a 20× objective. **(B)** The developmental stage of animals after 72 and 96 h of incubation from embryo at 16°C were determined and plotted, with a stacked bar chart indicating the relative proportion of larvae at each stage. **(C)** Log_2_ fold-change estimates of ribosomal protein levels in haploinsufficient RP mutants compared to stage-matched control animals are shown. Relative protein amounts were predicted by Differential Expression Proteomics (DEP package, R) based on three replicates of semi-quantitative proteomics analysis. **(D)** Body area measurements of stage-matched animals (at late L4 stage), normalized to the median body area of each wild type/balancer group are shown. Differences in the average normalized body area of mutant and control animals were determined using a two-tailed Welch’s two-sample *t* test. The central line represents the median and the bars represent interquartile distribution. **(E)** Brood size of each haploinsufficient RP mutant, normalized to the median of their respective wild-type controls is shown. Each dot represents the number of progeny per individual animal. Differences in the mean normalized brood size of each mutant, compared to its respective control, were analyzed using an independent two-tailed Student’s *t* test. Data distribution was assumed to be normal but this was not formally tested. **(F)** Lifespan of small subunit RP mutants (top) and large subunit RP mutants (bottom) alongside their respective wild-type controls are shown. Lifespan data are presented in a survival plot, analyzed using the Log-rank test and Bonferroni correction for multiple comparisons. Superscript numbers denote the specific wild type balancer chromosomes and are used to compare between an RP mutant and the wild-type counterpart. Balancer chromosomes are denoted as follows: +^1^ = *tmC20*, +^2^ = *tmC5*, +^3^ = *mIn1*. All experiments were performed in at least three biological replicates, and the animals were grown at 16°C. In panels C–F, RP mutants are color-coded for clarity: *rpl-33(0)/+*^*3*^ in green, *rpl-5(0)/+*^*3*^ in orange, *rps-10(0)/+*^*1*^ in blue, and *rps-23(0)/+*^*2*^ in red. The “*n*=” on the graphs indicates the total number of animals analyzed.

**Figure S1.**
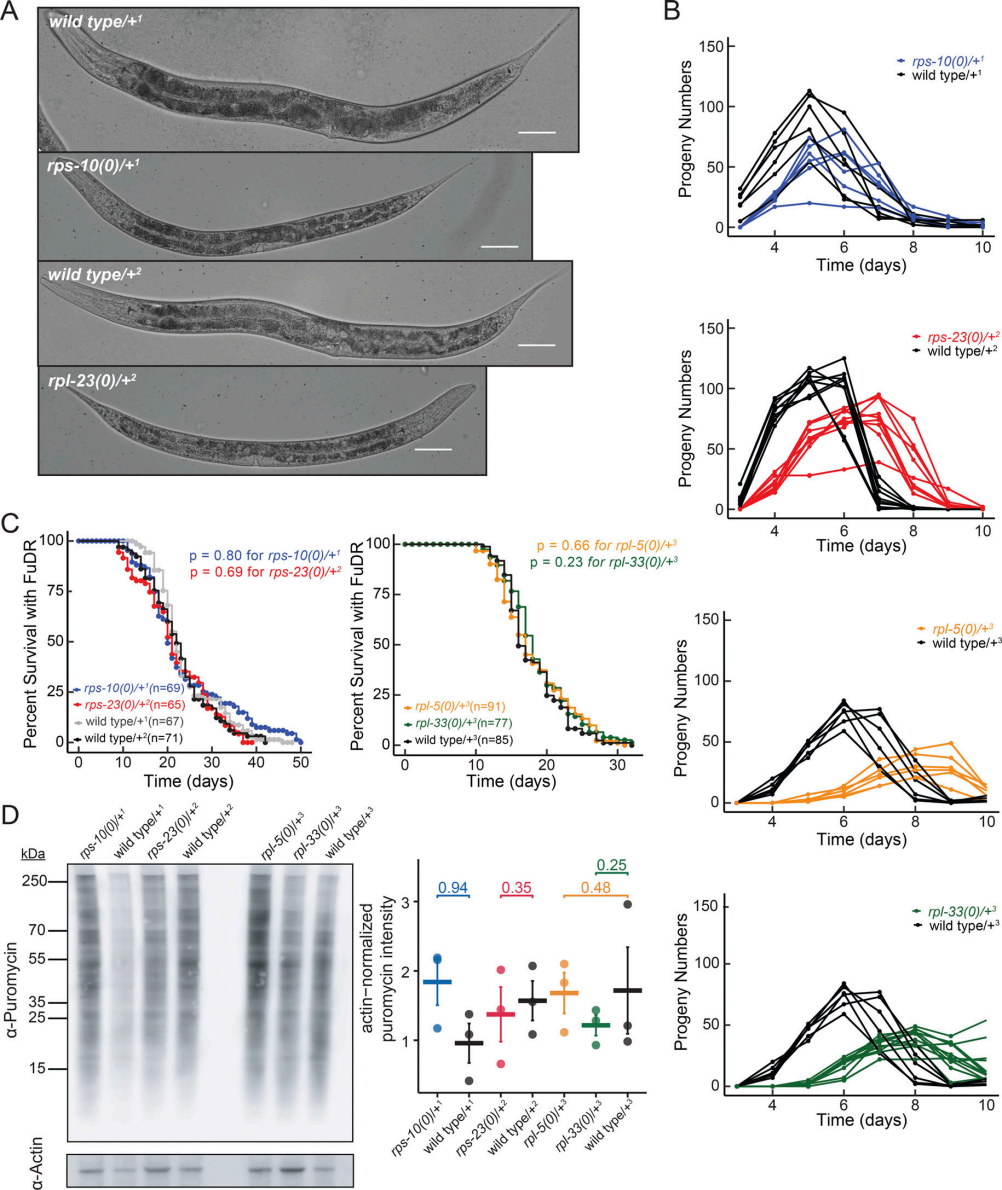
**Ribosomal protein haploinsufficiency results in developmental delays and reduced brood size without affecting lifespan in *C. elegans*. (A)** Development of small subunit RP haploinsufficient mutants and their respective wild-type counterparts are shown after 96 h of incubation from embryo at 16°C. Images depict a delay in growth and vulval development. Images taken with differential interference contrast, using a 20× objective. Scale bar represents 50 µm. **(B)** The brood size of RP mutants and their respective controls were counted and plotted with respect to time. Each line represents a single animal. **(C)** The lifespan of small subunit RP mutants (top) and large subunit mutants (bottom) were plotted along with their respective controls in the presence of FuDR (5-fluorodeoxyuridine). The y-axis represents percent survival and “*n*” represents the total number of animals. All experiments were done in three biological replicates with animals grown at 16°C. Statistical analysis was performed using Log-rank test with Bonferroni correction. Superscript numbers denote the specific balancers compared between an RP mutant and its wild type counterpart. Balancer chromosomes are denoted as follows: +^1^ = *tmC20*, +^2^ = *tmC5*, +^3^ = *mIn1*, +^4^ = *nT1*. **(D)** L4 worms were pulse-labeled with puromycin for 4 h. After labeling, equal amounts of protein were loaded for puromycin and actin Western blots, and puromycin levels were quantified and normalized to actin. Representative western blots for puromycin and actin are shown on the left. In the right-side plot, the y-axis represents actin-normalized puromycin intensity, with each point indicating a biological replicate. The median is marked by a horizontal line, and standard error is shown as a vertical line. For comparisons, each RP haploinsufficient sample was compared to its respective wild-type control using a one-tailed Student’s *t* test. Data distribution was assumed to be normal but this was not formally tested. All P values were >0.5, indicating that overall translation was not significantly reduced in stage-matched RP haploinsufficient animals. Source data are available for this figure: SourceData FS1 D.

We sought to determine the impact of haploinsufficiency of RP genes in *C. elegans* and focused on the single-copy losses of two large subunit RPs, *rpl-5* and *rpl-33,* along with two small subunit RPs, *rps-10* and *rps-23* (Cenik et al., 2019). We prioritized these four RP genes due to their involvement in human ribosomopathies, with *rpl-5*, *rpl-33*, and *rps-10* relating to DBA (Farrar et al., 2008), and *rps-23* relating to microcephaly and intellectual disability without the blood phenotypes (Paolini et al., 2017). Moreover, the protein products of these RP genes are incorporated into nascent ribosomes at different stages (nucleolar, nuclear, and cytoplasmic) (de la Cruz et al., 2015). We observed developmental delays across these RP haploinsufficient mutants compared with wild-type counterparts (Fig. 1, A and B; and Fig. S1 A). Protein levels were evaluated using semiquantitative proteomics against stage-matched controls, revealing reductions of ∼50% for RPL-33 and RPS-23, 25% for RPL-5, and 10% for RPS-10 (Fig. 1 C and Table S1). The developmental delays observed in haploinsufficient strains, as compared with their time-matched controls, were found to generally correspond with the degree of protein reduction resulting from the loss of a single copy (Fig. 1 C). These findings suggest a correlation between the extent of protein level reduction and the timing of developmental processes.

Similar to previous reports of reduced body size in RP mutants in other species (Marygold et al., 2007; Oliver et al., 2004), our examination revealed that, when given sufficient time to reach the same developmental stage, *C. elegans* RP haploinsufficient mutants were slightly smaller in body size than wild-type controls with one exception (Fig. 1 D). Specifically, we observed that *rps-10(0)/+* animals were slightly larger in body size compared with their stage-matched controls (Fig. 1 D, P = 0.02, independent Student’s *t* test). The increased body size could be associated with increased cell volume, altered cytoskeletal dynamics, or metabolism due to changes in signaling pathways such as TGF-β, MAPK, or cGMP (Hirose et al., 2003; Wang et al., 2002; Watanabe et al., 2005). These results are also reminiscent of the larger wing sizes and wing discs observed in *Drosophila* following the single copy loss of *RpL38* and *RpL5* (Marygold et al., 2005). However, why single copy loss of certain RP genes leads to increased organ or body growth remains unclear.

We further conducted fecundity assays to assess impacts on reproduction. Except for *rpl-5(0)/+* animals, which exhibited a significant reduction in progeny size (∼25% reduction; P = 0.03, independent Student’s *t* test), the progeny sizes of all other RP haploinsufficient mutants were similar to those of the controls (Fig. 1 E). The onset of peak fertility was delayed in all mutants except for *rps-10(0)/+* animals (Fig. S1 B). Additionally, RP mutants remained fertile for extended periods, thereby compensating for the overall progeny size, except for *rpl-5(0)/+* mutants. Reduced fertility was observed in *Drosophila minute* mutants, characterized by the lack of a single copy of an RP gene (Marygold et al., 2007). The observation of similar brood sizes in the majority of RP strains in *C. elegans* suggests the involvement of compensatory mechanisms within the germline.

Finally, we investigated lifespan in *C. elegans* RP mutants. Our lifespan analysis did not reveal significant differences for the majority of RP haploinsufficient mutants compared with wild-type controls, regardless of treatment with the egg-laying inhibitor fluorodeoxyuridine (FuDR). The only exception was *rps-23(0)/+* mutants that displayed a modest but significant extension of lifespan only in the absence of FuDR (Fig. 1 F and Fig. S1 C, P = 0.007 for *rps-23(0)/+* mutants, Log-rank test with Bonferroni correction). Reduced protein translation or the knockdown of RP genes is typically linked to increased lifespan (Syntichaki et al., 2007; Steffen et al., 2012; Rogers et al., 2011; Chiocchetti et al., 2007; Curran and Ruvkun, 2007; Tiku et al., 2017). However, our results suggest that haploinsufficiency for single RP genes negate the typical lifespan extension benefits associated with decreased protein synthesis due to imbalances in RP expression and the associated stress. To determine whether overall translation levels were reduced in response to RP haploinsufficiency, we quantified puromycin incorporation in stage-matched animals over a limited time period. Our results suggest that haploinsufficiency for single RP genes does not result in a significant reduction in overall translation when compared to stage-matched respective controls (Fig. S1 D, P value >0.05, one-tailed Student’s *t* test). Taken together, our phenotypic characterization of the haploinsufficiency of RP genes in *C. elegans* reveals a range of developmental and physiological consequences that broadly mimic those observed in other model organisms such as *Drosophila* and mice (Marygold et al., 2007; Oliver et al., 2004).

### Adaptive cellular responses to ribosomal protein loss highlights SKN-1–dependent enhanced oxidative stress resistance

**Figure 2.**
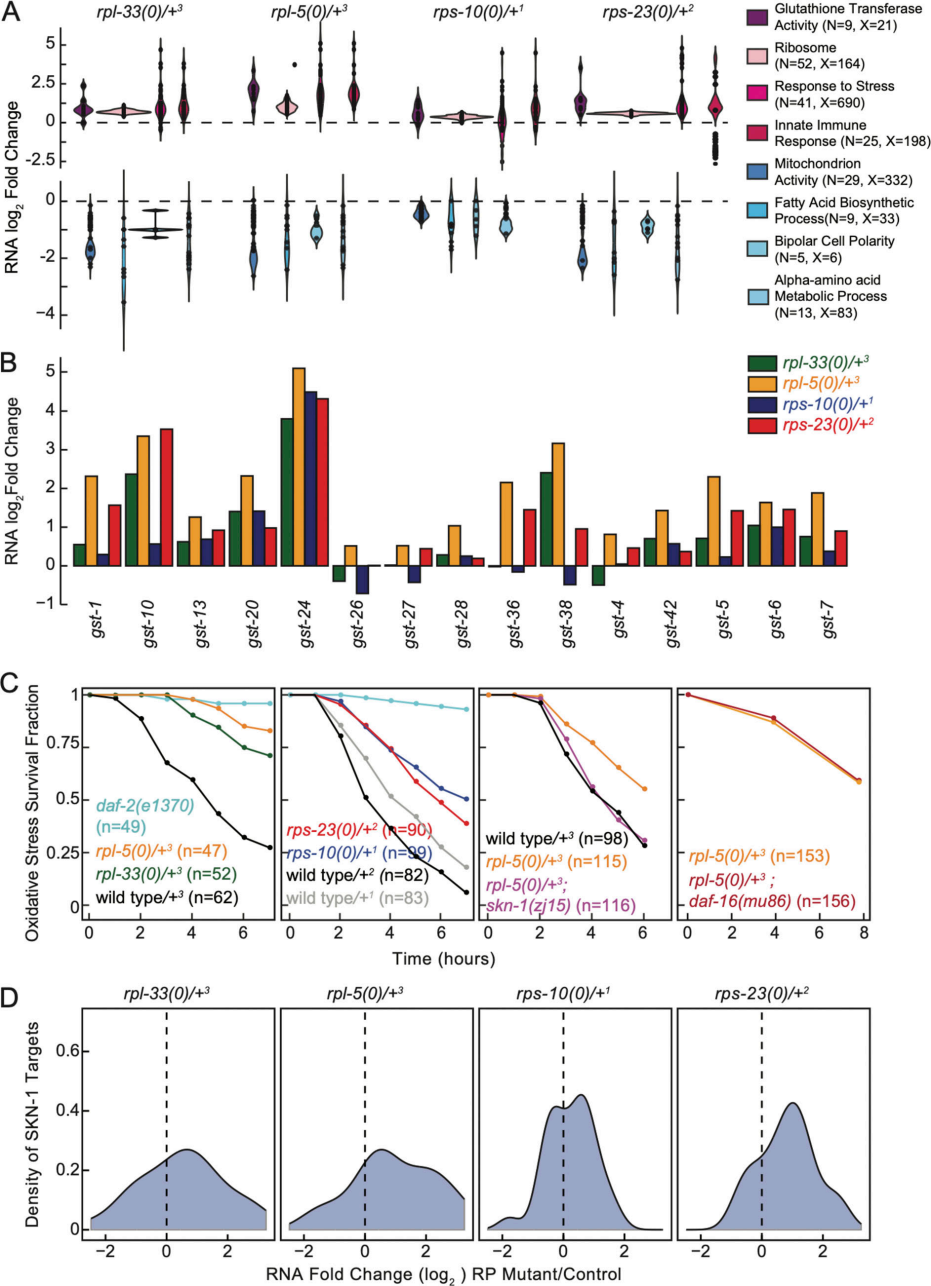
**Adaptive cellular responses to ribosomal protein loss highlights SKN-1 dependent enhanced oxidative stress resistance. (A)** Investigation of significantly differentially expressed genes at the RNA level across all haploinsufficient RP mutants, with a focus on significant gene ontology (GO) enrichments are shown. Log_2_ fold-change gene expression estimates of representative GO categories that were enriched for overexpression (top) and underexpression (bottom) were plotted. All significantly enriched GO categories are provided in Table S3. The y-axis displays predicted RNA-seq log_2_ fold change estimate distribution for each GO category as predicted by edgeR. GO categories are annotated on the side, where “*N*” denotes the number of differentially expressed genes within a category, and “X” represents the total number of genes in that category. Each dot signifies an individual gene identified as underexpressed or overexpressed, with their distribution across the categories visualized through violin plots. **(B)** RNA expression log_2_ fold-change estimates of glutathione transferase genes annotated in the *C. elegans* genome across all mutants compared with their controls. **(C)** An acute time course of oxidative stress survival for large subunit RP mutants (first plot, *rpl-5(0)/+* and *rpl-33(0)/+*) and small subunit RP mutants (second plot, *rps-23(0)/+* and *rps-10(0)/+*), alongside wild-type control strains and *daf-2(e13270)* mutants serving as positive controls. All RP mutants showed significantly more resistance to acute oxidative stress (P < 0.001) compared with wild-type controls. *rpl-5(0)/+*; *skn-1(zj15)* double mutant animals were significantly less resistant to oxidative stress compared with *rpl-5(0)/*mutants (P = 0.016, third plot). Conversely, *rpl-5(0)/+; daf-16(mu86)* animals displayed similar responses to oxidative stress as *rpl-5(0)/+* animals (P = 0.51, fourth plot). The “*n*=” on the graphs indicates the total number of animals analyzed. “*n*” indicates the total number of animals used in the study, and all experiments were performed using three biological replicates and all animals were grown at 16°C. *rpl-33(0)/+*^*3*^ in green, *rpl-5(0)/+*^*3*^ in orange, *rps-10(0)/+*^*1*^ in blue, and *rps-23(0)/+*^*2*^ in red. Balancer chromosomes are denoted as follows: +^1^ = *tmC20*, +^2^ = *tmC5*, +^3^ = *mIn1*. For statistical analysis of oxidative stress resistance, Log-rank analysis with Bonferroni correction was performed for multiple comparisons. **(D)** Log_2_ fold-change estimates of SKN-1 targets in all RP mutants were plotted. SKN-1 targets were identified from differentially expressed genes upon the constitutive activation of SKN-1 (Nhan et al., 2019).

To understand the cellular mechanisms triggered by single copy losses of RP genes in *C. elegans*, we performed RNA sequencing (RNA-seq) on stage-matched mutant and control animals at the L4 stage (Table S2). This analysis, which included stage-matched controls to mitigate any developmental delay effects, revealed a uniform gene expression response across all mutants. This response was characterized by overexpression of ribosomal machinery, glutathione transferase activity, and genes involved in innate immunity and stress responses, indicating a systemic adaptation to RP loss (Fig. 2 A, top, significantly enriched gene ontology [GO] categories provided in Table S3). Conversely, genes related to mitochondrial activity, fatty acid biosynthesis, cell polarity, and amino acid metabolism were significantly underexpressed (Fig. 2 A, bottom, Table S3), suggesting a reprogramming of cellular metabolism in response to RP haploinsufficiency.

Given the pronounced overexpression of glutathione transferase (*gst*) genes (∼2.8-fold enrichment, P < 0.001, GO enrichment), we assessed the expression patterns of *gst* genes across all RP mutants (Fig. 2 B). The general overexpression signature aligns with previously established links between glutathione transferase activity and oxidative stress resistance (Ayyadevara et al., 2005; Burmeister et al., 2008), prompting us to assess the mutants’ resilience to oxidative stress.

In acute survival assays using high doses of paraquat, we observed that all RP haploinsufficient strains exhibited significantly enhanced resistance to oxidative stress compared with wild-type controls (Fig. 2 C, first and second plot, P < 0.01, Log-rank test with Bonferroni correction). This widespread increase in stress resistance suggests a robust, adaptive mechanism that compensates for elevated ROS levels.

**Figure S2.**
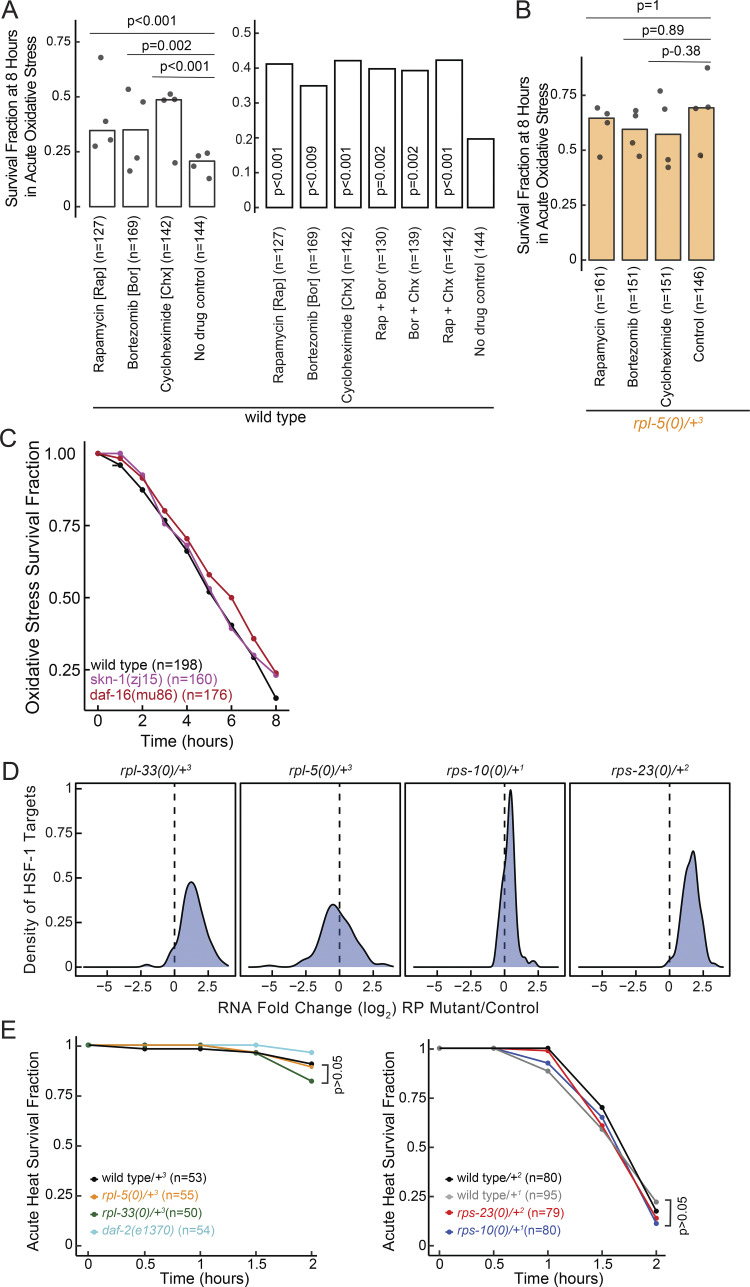
**Stress responses in RP haploinsufficient mutants. (A)** Survival fractions analyzed after 8 h of acute oxidative stress after pretreatment of rapamycin, bortezomib, and cycloheximide in wild-type animals are shown (left). Wild-type animals pretreated with double combinations of rapamycin, bortezomib, and cycloheximide and survival fraction within 8 h of acute oxidative stress was determined (right). **(B)** Survival fractions analyzed after 8 h of acute oxidative stress after pretreatment of rapamycin, bortezomib, and cycloheximide in *rpl-5(0)/+* mutants are shown. In A and B each point represents a biological replicate and bars represent the median of all biological replicates. Statistical analysis was performed using Log-rank test with Bonferroni correction. P values are provided on the top of the plot. For the right plot in A all samples were compared to no drug control. The “*n* = ” on the graphs or legends indicates the total number of animals analyzed. **(C)** An acute oxidative stress survival time course for *skn-1(zj15)* and *daf-16(mu86)*, alongside wild-type animals, is shown. *skn-1(zj15)* and *daf-16(mu86)* animals are comparable in acute oxidative stress survival compared to wild-type animals (P values are 0.52 and 0.08 for *skn-1(zj15)* and *daf-16(mu86)* respectively). (P = 0.85 for comparison of *skn-1(zj15)* versus *daf-16(mu86)*). P values were obtained using a Log-rank test with Bonferroni correction to compare the survival distributions of two or more groups for three biological replicates. **(D)** Density plots of log_2_ RNA expression differences of HSF-1 targets across all RP haploinsufficient mutants were plotted. HSF-1 targets are the significantly overexpressed genes in response to HSF-1 overexpression (Sural et al., 2019). **(E)** Acute heat stress survival was assessed in RP mutants, balancer controls, and *daf-2(e1370)* heat-stress-tolerant mutants after 2 h at 37°C. Statistical analyses were performed using Log-rank test, with Bonferroni correction for multiple samples. All experiments were conducted in three biological replicates, all animals were grown at 16°C. “*n*” in A–C represents the number of animals. Superscript numbers denote the specific balancers compared between an RP mutant and its wild type counterpart. Balancer chromosomes are denoted as follows: +^1^ = *tmC20*, +^2^ = *tmC5*, +^3^ = *mIn1*.

Perturbations in ribosome biogenesis have been shown to elicit proteotoxicity (Tye et al., 2019). Moreover, RP haploinsufficiency reduces ribosome levels (Khajuria et al., 2018), which could lead to a decrease in overall protein synthesis. To determine whether the observed elevated levels of oxidative stress in the mutants were due to proteotoxic stress or were related to a reduction in protein synthesis, we pretreated wild-type animals with inhibitors targeting key pathways: the proteasome (bortezomib), ribosome biogenesis and the translation regulator TORC1 (rapamycin), and translation elongation (cycloheximide) before assessing survival under acute oxidative stress conditions. These treatments significantly enhanced the stress response of wild-type animals (P < 0.05 for each drug, Log-rank test with Bonferroni correction), supporting the role of these pathways in mediating elevated oxidative stress resistance (Fig. S2 A, left panel). Moreover, the combined use of the inhibitors did not further improve survival rates in wild-type animals (Fig. S2 A, right panel). Finally, none of the treatments altered the survival outcomes of *rpl-5(0)/+* mutants under oxidative stress (P ≥ 0.4, Log-rank test with Bonferroni correction), suggesting that *rpl-5(0)/+* mutants inherently possess an elevated baseline oxidative stress response (Fig. S2 B).

Unfolded protein and oxidative stress responses are mediated through SKN-1, which is orthologous to human NRF2 (Glover-Cutter et al., 2013; Oliveira et al., 2009; Xu et al., 2018; Hu et al., 2017; Inoue et al., 2005). SKN-1 further induces a transcriptional response that results in stress resistance when protein translation is inhibited (Wang et al., 2010). Moreover, TORC1 signaling pathway and rapamycin regulate both SKN-1 and DAF-16, orthologous to human FOXO3 (Robida-Stubbs et al., 2012). Additionally, DAF-16 is involved in the repression of RP genes, serving for resistance to hypoxia resistance (Hemphill et al., 2022). Thus, we hypothesized that SKN-1 and DAF-16 might be regulators of the oxidative stress response observed in RP mutants. To dissect these regulatory pathways, we evaluated the oxidative stress survival of *rpl-5(0)/+* mutants in combination with mutations in *skn-1* and *daf-16* genes. The *skn-1(zj15)* hypomorphic mutation (Tang et al., 2016) in the *rpl-5(0)/+* mutant background significantly diminished oxidative stress survival (Fig. 2 C, third plot, P = 0.02, Log-rank test), suggesting that SKN-1 is essential for the resistance of oxidative stress in *rpl-5(0)/+* mutants. *skn-1(zj15)* mutants were not resistant to oxidative stress suggesting that SKN-1 is likely chronically activated in *rpl-5(0)/+* mutants (Fig. S2 C, P = 0.52, Log-rank test with Bonferroni correction). We did not observe a significant survival change in *rpl-5(0)/+* mutants when crossed with the *daf-16(mu86)* mutation (Lin et al., 1997), highlighting SKN-1’s unique contribution (Fig. 2 C, fourth plot, P = 0.5, Log-rank test). *daf-16(mu86)* mutants did not display differential resistance to acute oxidative stress compared with wild-type controls (Fig. S2 C, P = 0.08, Log-rank test with Bonferroni correction). We also observed a mild overexpression of genes upregulated in response to *skn-1* gain of function across the RNA-seq datasets for RP haploinsufficient mutants (Fig. 2 D [Nhan et al., 2019]). This finding suggests that SKN-1 activity may not be limited to *rpl-5(0)/+* mutants but likely extends to other RP mutants as well.

In *Saccharomyces cerevisiae*, aberrations in ribosome biogenesis lead to the upregulation of targets of Hsf1, a key regulator of proteotoxic stress response (Li et al., 2017; Tye et al., 2019; Albert et al., 2019). To investigate whether a similar pattern occurred in our RP haploinsufficient mutants, we examined the expression levels of genes that are known targets of HSF-1 overexpression (Sural et al., 2019). Our analysis indicates a trend toward overexpression of HSF-1-regulated genes in two of the mutants, *rpl-33(0)/+* and *rps-23(0)/+* (Fig. S2 D). However, the overexpression of HSF-1 targets did not translate into observable differences in acute heat resistance over a 2-h period (Fig. S2 E, P > 0.05). Notably, in *C. elegans*, the responses to oxidative stress and heat stress act in opposition and attenuate each other’s effects (Crombie et al., 2016). Taken together, these observations suggest that while there may be alterations in the expression of HSF-1 targets under RP haploinsufficiency, this does not necessarily indicate activation of HSF-1 or enhanced stress tolerance. It may point to a complex interplay in the cellular stress response which requires further investigation.

### Translational regulation ensures balanced ribosomal expression despite haploinsufficiency

**Figure S3.**
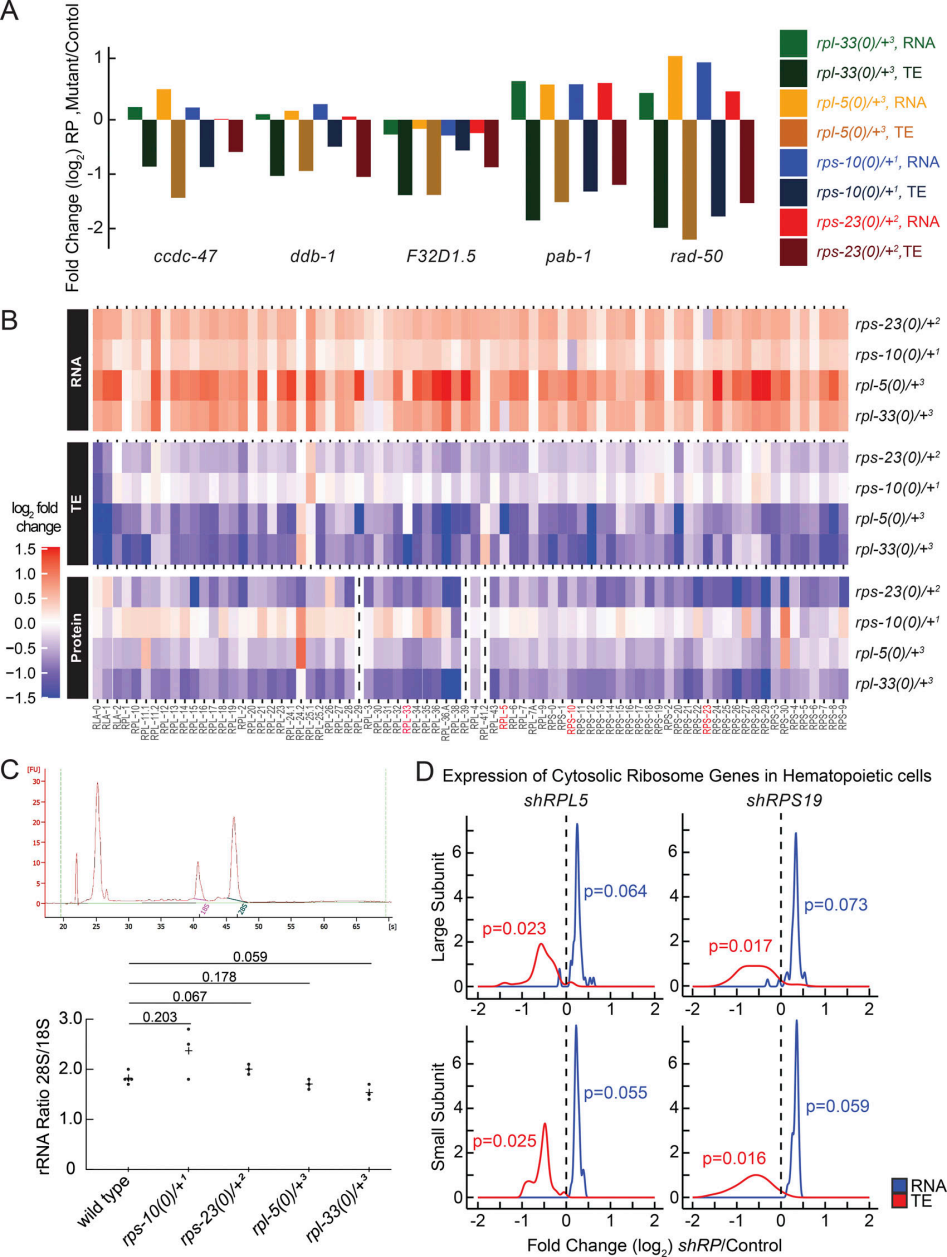
**Gene expression and translation efficiency (TE) differences in RP haploinsufficient mutants. (A)** RNA and TE log_2_ ratio estimates for the top five most significant genes for all RP haploinsufficient mutants were plotted. The y-axis shows the log_2_ fold change predictions, with TE and RNA levels for each mutant labeled in distinct colors. Superscript numbers denote the specific balancers compared between an RP mutant and its wild type counterpart. Balancer chromosomes are denoted as follows: +^1^ = *tmC20*, +^2^ = *tmC5*, +^3^ = *mIn1*, +^4^ = *nT1*. **(B)** Heatmap depicting the log_2_ fold change of each RP gene expression at the RNA, TE, and protein levels for all four different mutants. The heatmap scale on the left represents non-scaled log_2_ fold ratio estimates. **(C)** The 28S/18S rRNA ratio was calculated based on area measurements from Bioanalyzer RNA results. The top chart represents an example of a Bioanalyzer result. In the bottom plot, the y-axis shows the 28S/18S ratios, with each point representing a biological replicate. Each RP haploinsufficient mutant sample was compared to stage-matched wild-type control sample using a Student’s *t* test. All P values were >0.5, indicating no significant differences in the ratios were observed. Data distribution was assumed to be normal but this was not formally tested. **(D)** RNA expression and TE differences were plotted along the y-axis for all ribosomal protein genes that belong to large subunit (top) and small subunit (bottom) were plotted for *shRPL5* and *shRPS19* knockdown in hematopoietic cells (Khajuria et al., 2018). For statistical analysis of changes in expression of large and small subunit ribosomal protein genes at RNA and TE level, ROAST multivariate gene expression analysis was conducted (Wu et al., 2010).

To investigate the impact of single-copy RP gene loss on translation, we used ribosome profiling (Ribo-seq) alongside RNA-seq to analyze stage-matched mutant and control *C. elegans* animals at the L4 developmental stage. This approach allowed us to identify translational efficiency (TE) alterations across four RP mutants (Table S4). Notably, genes such as *ccdc-47*, *ddb-1*, *F32D1.5*, *pab-1*, and *rad-50* exhibited significantly decreased TE across all RP mutants (p_adj_ < 0.05), with *pab-1* and *rad-50* also showing RNA overexpression, hinting at a potential compensatory mechanism in response to reduced TE (Fig. S3 A).

**Figure 3.**
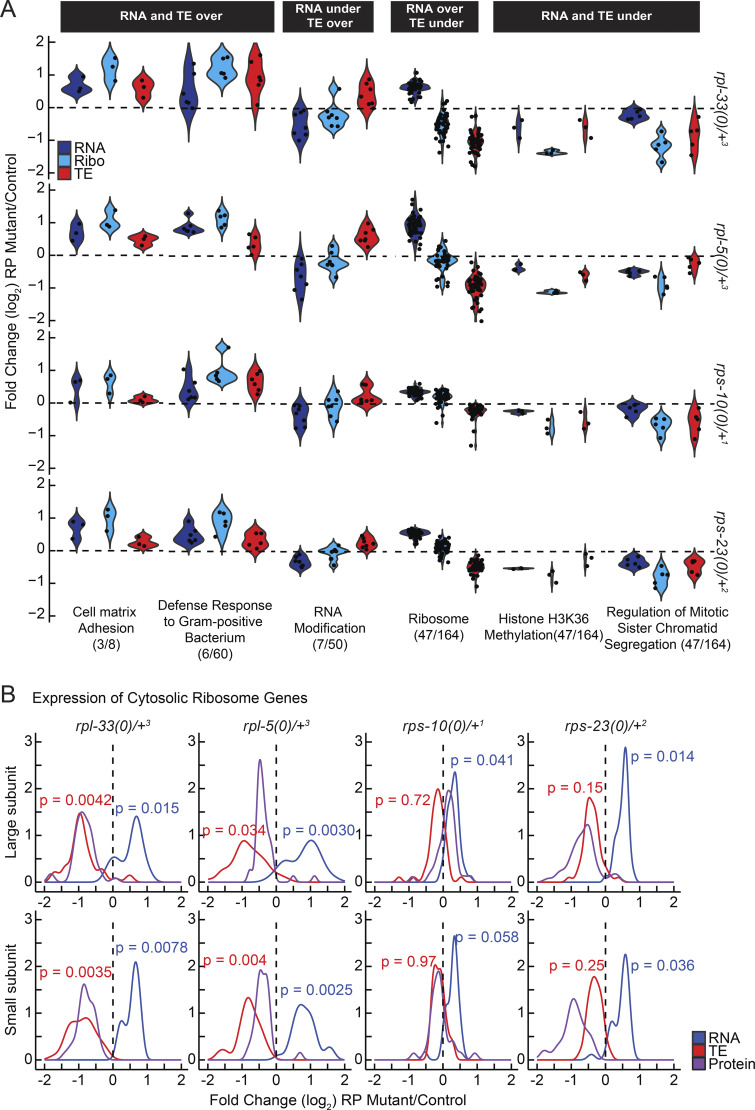
**Translational regulation ensures ribosome balance despite haploinsufficiency. (A)** Genes were filtered based on log_2_ fold-change estimates from RNA-seq, Ribo-seq, and translation efficiency (TE), using the following criteria: (1) over or under expression at both the RNA and TE levels across all mutants, and (2) expression changes in opposite directions at the RNA or TE levels, indicating potential buffering effects. Significant gene ontology (GO) enrichment was performed for each of these categories (upper black panels: RNA and TE over, RNA under TE over, RNA over TE under, RNA and TE under). Log_2_ fold-change distributions from edgeR predictions were plotted for selected significant GO categories, showing RNA-seq levels (blue), Ribo-seq levels (Ribo, cyan), and TE levels (TE, red). Significant GO annotations are provided below (X/N), where X denotes the number of significantly expressed genes and *N* represents the total number of genes in the GO category. Each dot in the plot represents a gene differentially expressed in the significant GO category annotated below. **(B)** Density plots of log_2_ fold change estimates (RP mutant/control) in RNA, TE, and protein expression for all detected ribosomal protein (RP) genes (large or small subunit) in response to the single-copy loss of each RP haploinsufficient mutant, relative to stage-matched control animals generated. The density lines represent different data types: blue for RNA, red for TE and purple for protein levels. Each RP haploinsufficient mutant genotype is labeled at the top of the plots. The y-axis represents large subunit RP genes in the top row and small subunit RP genes in the bottom row. The x-axis displays the log_2_ fold-change estimate distributions for RP haploinsufficient/control comparisons. ROAST multivariate gene expression analysis was conducted to calculate P values for RNA and TE level expression differences in small and large subunit RP gene expression. P values provided on the plots (blue and red values represent RNA TE level differences respectively) and indicates significant overexpression of both small and large subunit RP genes at the RNA level in all mutants, while showing significantly decreased TE levels for both subunits across all RP haploinsufficient mutants, except for *rps-10(0)/+*^*1*^. Log_2_ fold changes in *rps-10(0)/+*^*1*^ are smaller in magnitude, likely due to the relatively modest decrease in RPS-10 protein levels (∼10% decrease, Fig. 1 C).

Further analysis revealed distinct expression trends. Cell matrix adhesion and defense response genes were overexpressed at both RNA and TE levels (Fig. 3 A, “RNA and TE over,” Table S4, significantly enriched GO category list provided in Table S5). In contrast, genes involved in histone H3K36 methylation and sister chromatid segregation were underexpressed at both levels, suggesting a systematic downregulation in these functional categories (Fig. 3 A, “RNA and TE under,” Tables S4 and S5). Given the involvement of H3K36 methylation in processes such as RNA polymerase II–mediated elongation (Carrozza et al., 2005) and the regulation of alternative splicing (Luco et al., 2010), a reduction in the expression of components of this pathway could further contribute to alterations in transcription and the diversity of transcript isoforms being produced.

Interestingly, ribosomal component genes were overexpressed at the RNA level but had reduced TE, notable in both large and small subunit genes (Fig. 3 A, “RNA under TE over” and “RNA over TE under,” respectively, Tables S4 and S5). This differential regulation highlights a complex response that balances transcription and translation in response to RP haploinsufficiency to maintain overall ribosome numbers.

In *S. cerevisiae*, mutations in small subunit genes do not impact the expression of large subunit genes, resulting in an accumulation of unpaired large subunits (Cheng et al., 2019). In contrast, our observations in *C. elegans* indicate a coordinated response across both subunits at the RNA and TE levels: reductions in RPs from either subunit led to a generalized overexpression at the RNA levels (P ≤ 0.01 except *rps-10(0)/+*, ROAST–rotation gene set testing [Wu et al., 2010]), but a decrease in TE levels (P ≤ 0.05 for *rpl-33(0)+* and *rpl-5(0)/+*, ROAST) (Fig. 3 B and Fig. S3 B). To assess the potential accumulation of unpaired subunits, we analyzed 28S/18S ratios by measuring the area underneath 28S and 18S peaks in total RNA samples from RP haploinsufficient and wild-type animals using Bioanalyzer. We did not observe significant changes in this ratio (Fig. S3 C, P > 0.05; RP haploinsufficient mutants were compared to wild-type controls using Student’s *t* test with Bonferroni correction). Given that such a response was not observed in yeast, we asked whether a similar response occurs in response to DBA-specific RP gene reductions in human hematopoietic cells (Khajuria et al., 2018). Reanalyzing the RNA-seq and Ribo-seq datasets from this study revealed that the results in *C. elegans* mirror those from human *RPL5* and *RPS19* knockdowns in hematopoietic cells, where TE of all subunits are significantly decreased (Fig. S3 D, P < 0.05, ROAST, Table S6). Hence, the human cellular response is more similar to that in *C. elegans*, in comparison to yeast, highlighting a potentially conserved regulatory mechanism regulating translation for maintaining ribosome production under conditions of single-copy loss of RP genes.

### Mitochondrial translation and morphology differences in response to ribosomal protein haploinsufficiency

In RP haploinsufficient mutants, we observed upregulation of glutathione transferase activity and potential activation of SKN-1, a critical factor in maintaining cellular redox balance and facilitating mitochondrial retrograde signaling (Palikaras et al., 2015; Dinkova-Kostova and Abramov, 2015). These observations suggest a potential impairment in mitochondrial function. In particular, genes of the electron transport chain (ETC) require coordinated translation by both cytoplasmic and mitochondrial ribosomes, creating a vulnerability in proteostasis (Soto et al., 2022).

**Figure 4.**
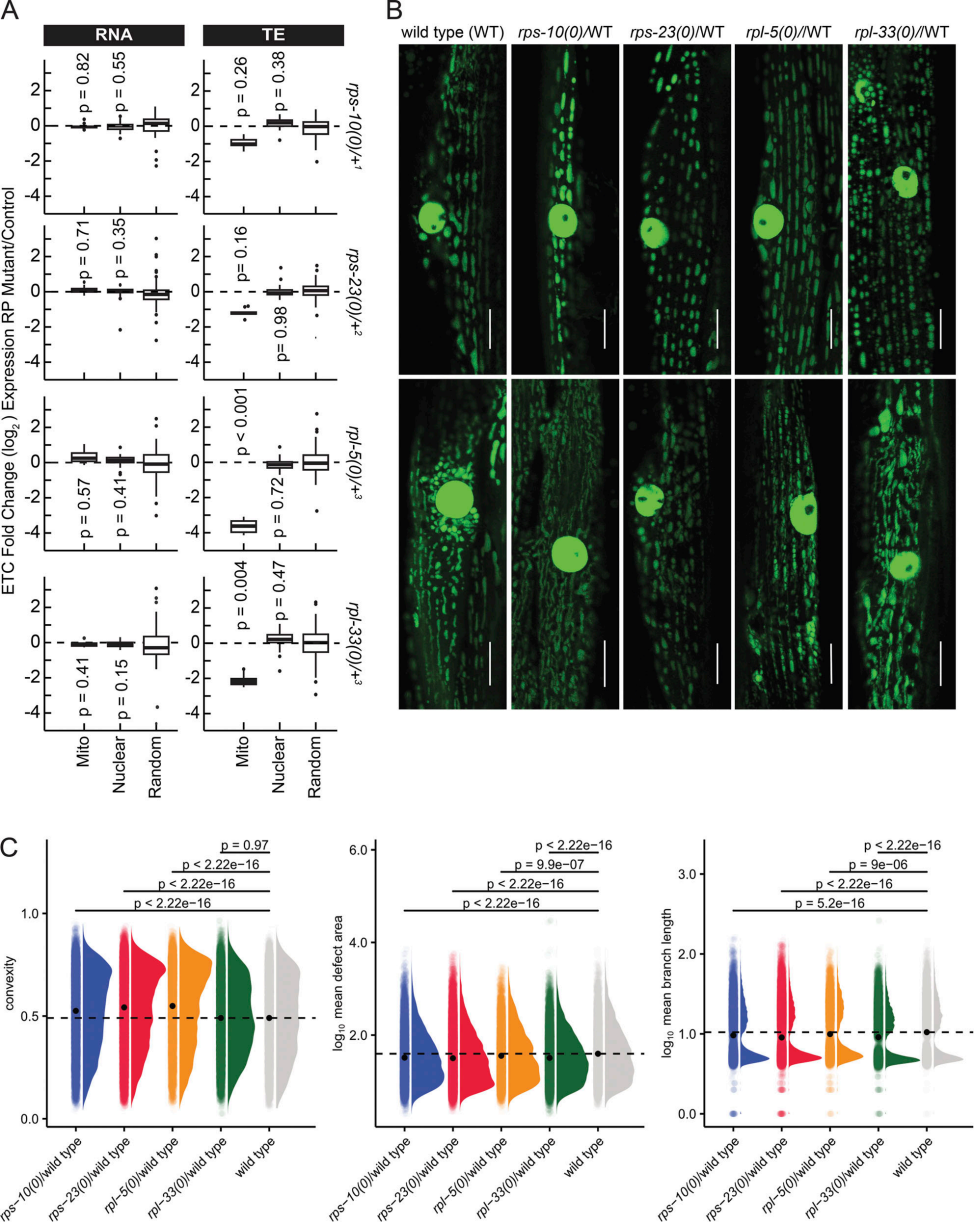
**Mitochondrial translation and morphology in response to RP haploinsufficiency in *C. elegans*. (A)** Log_2_ fold change estimates in RNA and translation efficiency (TE) levels were plotted along the y-axis for mitochondrial-encoded (mito) or nuclear-encoded (nuclear) electron transport chain (ETC) genes, as well as for a randomly selected gene set, for each RP haploinsufficient mutant. Log_2_ fold changes for RNA and TE were plotted on the left and right sections, respectively, and are labeled at the top. Each row represents a different RP haploinsufficient mutant, with the genotype labeled on the right-most side. On the x-axis, “mito” and “nuclear” represent mitochondrially and nuclear-encoded ETC component genes, respectively. The “random” set represents a group of random genes, equal in size to the nuclear-encoded ETC component gene set. For statistical comparison of RNA and TE expression differences in mitochondrial or nuclear-encoded ETC genes. ROAST multivariate gene expression analysis was conducted (Wu et al., 2010). The P values derived from the ROAST analysis are indicated on each box plot. According to this analysis, mitochondrially encoded transcripts are translated significantly less in *rpl-5(0)/+* and *rpl-33(0)/+* mutants (P < 0.005). While the overall distribution is lower in *rps-10 (0)/+* and *rps-23(0)/+*, the changes are more modest and not statistically significant (P > 0.05). Superscript numbers denote the specific wild type balancer chromosomes and are used to compare an RP mutant and the wild-type counterpart. Balancer chromosomes are denoted as follows: +^1^ = *tmC20*, +^2^ = *tmC5*, +^3^ = *mIn1*. **(B)** Representative mitochondrial morphology images using mitochondrial and nuclear-localized GFP in body wall muscle cells in RP mutants as well as stage-matched wild-type controls. Day 3 adult animals, which were transferred from 16°C to 23°C on the last 2 days were imaged. RP mutants in this analysis do not have a balancer chromosome. Images were taken with Stellaris Confocal with a 63× objective, and the scale bar represents 10 µm. **(C)** Distribution of mitochondrial morphological measurements of mutant and control animals. GFP signal localized to mitochondria was used to identify mitochondrial objects. Each mitochondrion was measured using convexity (degree to which shape differs from its convex hull), defect area (area outside of convex hull), and skeleton branch length. Mean measurements are represented as points, and the black dashed line corresponds to the wild-type mean. A two-sided Welch’s two-sample *t* test was used to compare differences in the mean measurement across each mutant relative to the wild type. Experiments in B and C were conducted with three biological replicates, analyzing multiple body wall muscle cells from at least nine animals per group (with >10^3^ mitochondria measurements per strain). Two mitochondria images per mutant and wild type were shown in B to represent variability of mitochondrial morphology per body wall cell.

**Figure S4.**
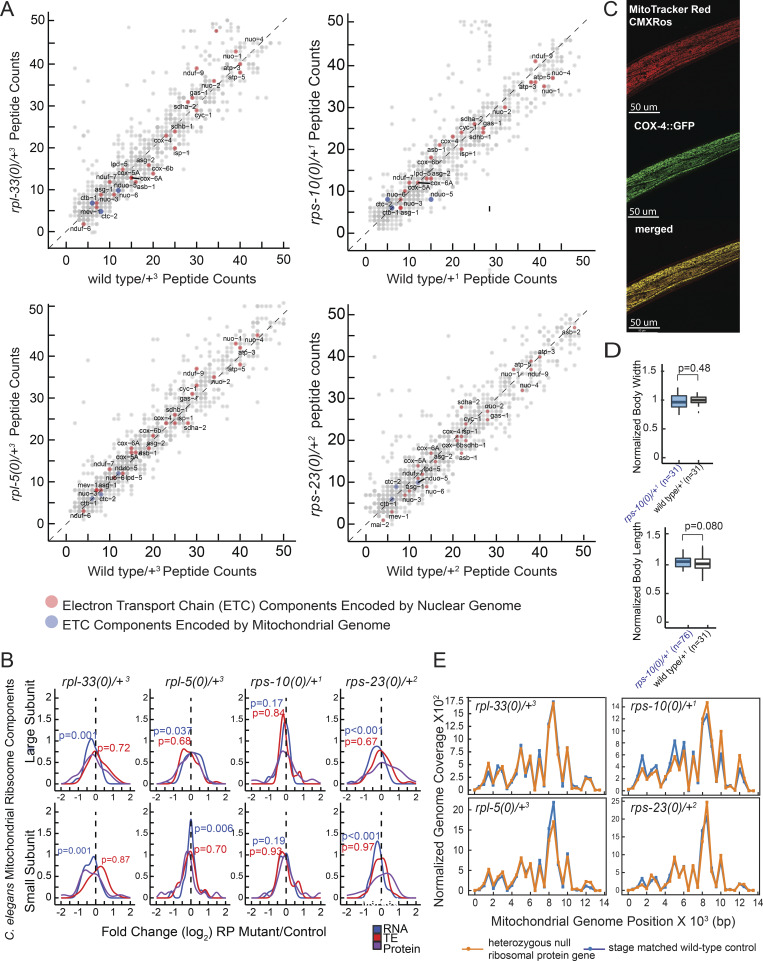
**Analysis of electron transport chain (ETC) component peptide counts, mitochondrial ribosome expression, mitoTracker CMXRos staining and mitochondrial abundance and DNA coverage in haploinsufficient RP mutants. (A)** Raw peptide counts from all three replicates were combined to analyze the ETC components, differentiating between those encoded by nuclear genes (red) and those encoded by mitochondrial genes (blue). Although coverage of mitochondrially encoded ETC components was lower, nuclear-encoded components showed a nearly diagonal pattern, indicating minimal deviation from expected levels. The y-axes of all four graphs display peptide reads from heterozygous ribosomal protein mutants, while the x-axes correspond to stage-matched wild-type controls. **(B)** Overall, RNA expression, TE, and protein level changes of all mitochondrial RP genes were plotted in RP haploinsufficient mutants. Y-axes show mitochondrial ribosomal proteins that belong to large or small subunits (top and bottom plots, respectively) and x-axes show log_2_ fold changes that were predicted by EdgeR for RNA and TE, and by DEP for proteins. For statistical analysis, ROAST multivariate gene expression analysis was conducted (Wu et al., 2010). **(C)** Staining specificity was assessed through co-localization studies using a *C. elegans* strain with a CRISPR-engineered knock-in of *cox-4* gene tagged with GFP (*cox-4::GFP*), serving as a marker for mitochondrial inner membranes. These co-localization analyses were conducted using a Leica Stellaris Confocal System equipped with a 63× objective. A representative image is shown with MitoTracker CMXRos staining (left), COX::GFP (middle) and merged images (right). Yellow color indicates co-localization of the staining with the mitochondrial inner membrane marker, COX-4. **(D)** Body length and width were assessed for stage-matched *rps-10(0)/+* and wild-type controls used in oxygen consumption experiments depicted in Fig. 5 C. **(E)** Mitochondrial genome coverage was charted for heterozygous *C. elegans* mutants and stage-matched wild-type controls. The x-axes represent mitochondrial genome positions, and the y-axes show mitochondrial genome coverage normalized to the nuclear genome. Orange lines indicate heterozygous mutant animals, and blue lines represent stage-matched wild-type controls at the L4 stage. Source data are available for this figure: SourceData FS4 C.

We next compared changes in RNA expression and TE of ETC components across RP mutants. While we did not observe significant changes in the expression of nuclear-encoded or mitochondrially encoded ETC components at the RNA level (Fig. 4 A, left, P > 0.1, ROAST), a distinct pattern emerged at the TE level. Nuclear-encoded components remained unaffected (P > 0.4), whereas mitochondrial-encoded ETC components showed reduced TE across all mutants (Fig. 4 A, right, P < 0.005 for *rpl-33(0)/+* and *rpl-5(0)/+* and P ≤ 0.16 for *rps-10(0)/+* and *rps-23(0)/+*, ROAST). This discrepancy suggests that the impaired mitochondrial translation may indirectly affect the stoichiometry of ETC. Although proteomics-level measurements lacked sufficient coverage to quantify corresponding changes in protein abundance for ETC components, the mitochondrially encoded Complex-I component, NDUO-5, was notably underexpressed in *rps-10(0)/+* animals (∼70% reduction, P_adj_ = 0.2, Fig. S4 A and Table S1).

Given the marked reduction in the TE of mitochondrial-encoded ETC components across all mutants, we investigated if there was a corresponding change in the abundance of mitochondrial ribosomes. Mitochondrial RP mRNAs were generally mildly reduced (P < 0.05 for *rpl-33(0)/+*, *rpl-5(0)/+ and rps-23(0)/+*; P < 0.2 for *rps-10(0)/+*, ROAST); however, no consistent changes were observed at the TE levels (Fig. S4 B, P > 0.5 for all mutants). Similarly, changes at the protein level were not consistent with a wider distribution of mitochondrial RPs in *rps-23(0)/+* animals (Fig. S4 B). These results suggest that the observed variations in TE across ETC components may be due to impaired mitochondrial translation rather than differences in mitochondrial ribosome abundance.

The trend of elevated expression of *gst* genes and SKN-1-mediated oxidative stress regulation, both indicative of increased ROS, alongside reduced TE of mitochondrially encoded ETC components in RP mutants, pointed toward potential mitochondrial dysfunction. Previous studies on mitochondrial dynamics established a link between mitochondrial dysfunction and morphological changes, particularly under stress (Zemirli et al., 2018). Specifically, fission-induced mitochondrial fragmentation, characterized by round mitochondria as opposed to networked mitochondria, is associated with increased ROS and elevated oxidative stress (Yu et al., 2006; Qi et al., 2011).

To investigate the impact of RP haploinsufficiency on mitochondrial morphology, we introduced a body-wall-specific nuclear and mitochondrial GFP marker into the backgrounds of RP haploinsufficient mutants (Ahnn and Fire, 1994). No differences were observed at 16°C up to the L4 stage, but upon transferring the L4 animals to 23°C and imaging by day 3 of adulthood, we detected partially penetrant increases in mitochondrial fragmentation across all mutants (Fig. 4 B). To quantify fragmentation, we measured the convexity (the degree to which shape differs from its convex hull), defect area (area outside of convex hull) and skeleton branch length of each individual mitochondria. Our results reveal that on average, mitochondria from *rps-10(0)/+*, *rps-23(0)/+*, and *rpl-5(0)/+* are significantly more convex, or less networked, compared with wild-type control animals (Fig. 4 C, first plot, P < 0.001, Student’s *t* test). Similarly, all four mutants had a significant average decrease in mean defect area and branch length relative to wild-type control animals (Fig. 4 C, second and third plots, P < 0.001, Student’s *t* test). Together, these results suggest that RP mutants have increased mitochondrial fragmentation that may indicate underlying mitochondrial dysfunction that is both temperature-sensitive and age-related. These results suggest that RP mutants struggle to maintain mitochondrial homeostasis, particularly under moderately higher temperatures, pointing toward a vulnerability in their ability to adapt to environmental conditions.

### Mitochondrial function is compromised in *rps-10(0)/+* mutants

The changes in mitochondrial morphology among RP mutants prompted us to investigate mitochondrial function. Given the link between mitochondrial structure and metabolism (Westermann, 2010), we next evaluated mitochondrial membrane potential using MitoTracker Red CMXRos staining (Sarasija and Norman, 2018) (Fig. S4 C) and analyzed the overall energy status of the animals by measuring their relative ADP/ATP ratios (Palikaras et al., 2015).

**Figure 5.**
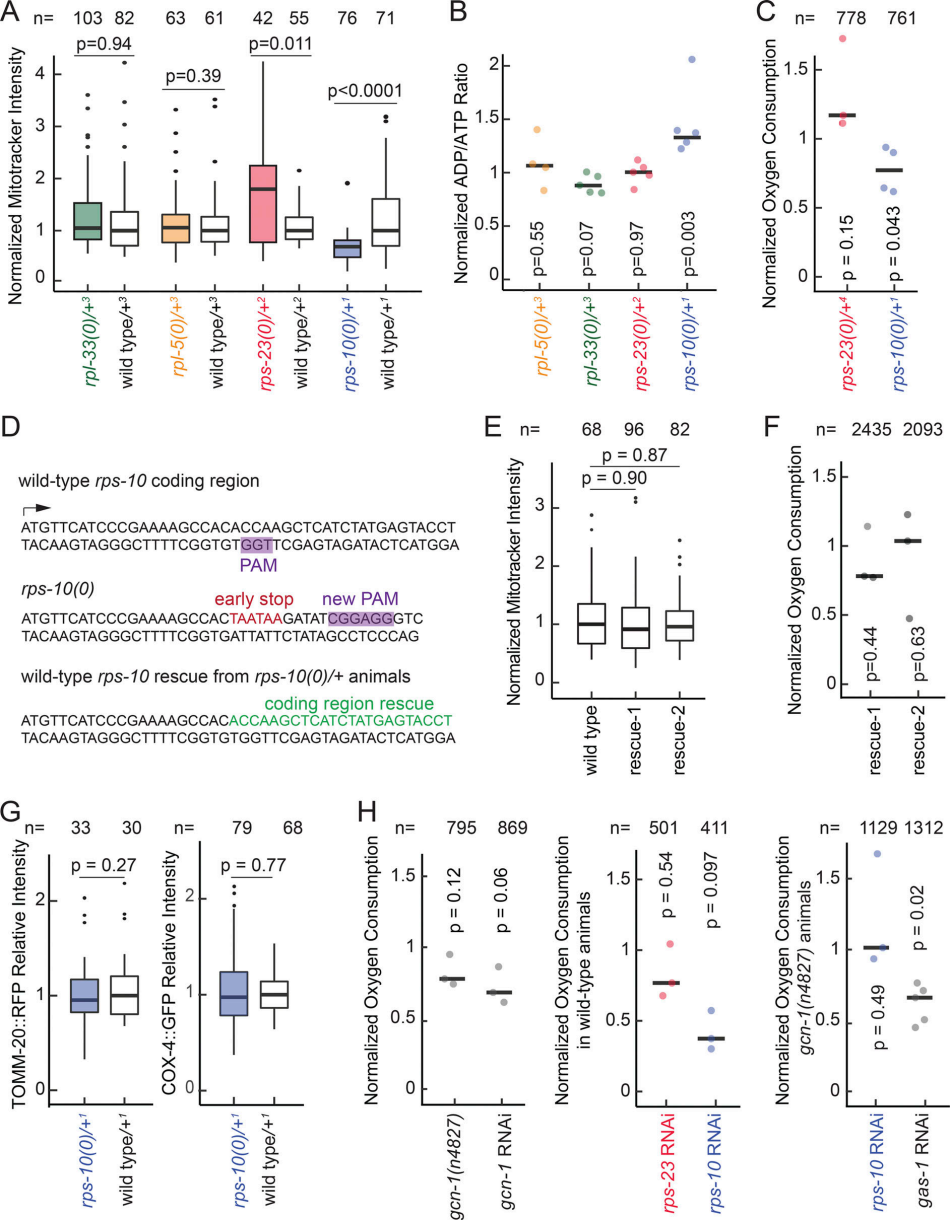
**Mitochondrial function and energy levels are compromised in *rps-10(0)/+* mutants. (A)** Mitochondrial activity, quantified via the uptake of fluorescent MitoTracker Red CMXRos dye in each RP mutant compared to its wild-type counterpart. MitoTracker intensity per area was measured, and the values for RP animals were normalized to the median of stage-matched wild-type controls and plotted. P values represent the results of a two-tailed Student’s *t* test with Bonferroni correction comparing each RP haploinsufficient mutant sample to its stage-matched wild-type control. Data distribution was assumed to be normal but this was not formally tested. **(B)** The ADP/ATP ratio, reflecting overall energy levels of RP haploinsufficient animals normalized to stage-matched controls is shown for each mutant. Measurements were taken from ∼50 RP mutant and wild-type animals per biological replicate, with each replicate represented as a single dot on the plot. **(C)** The relative oxygen consumption of *rps-23(0)/+* and *rps-10(0)/+* mutants compared to wild type was plotted. Animals were stage-matched to ensure similar body sizes, and oxygen consumption rates were normalized to the number of animals utilized in each assay. In B and C each point reflects the ratio of RP haploinsufficient animals to respective wild-type controls per biological replicate. The bold line represents the median. P values represent the results of a paired, two-tailed Student’s *t* test with Bonferroni correction comparing each RP haploinsufficient mutant to its stage-matched wild-type control. Data distribution was assumed to be normal but this was not formally tested. **(D)** The genetic modification strategy for the *rps-10* gene is illustrated. The wild-type *rps-10* gene with the PAM sequence highlighted in purple is shown on top; the introduction of tandem early stop codons, generating the *rps-10(0)* variant with a new PAM sequence in purple is indicated in the middle; the restoration of *rps-10(0)* to its wild-type sequence marked in green is demonstrated at bottom. **(E)** The normalized MitoTracker intensity was compared in wild type and two [*rps-10(0)* to wild-type] rescued strains (rescue-1, rescue-2). P values represent the results of a two-tailed Student’s *t* test with Bonferroni correction comparing each rescue strain to the stage-matched wild-type controls. Data distribution was assumed to be normal but this was not formally tested. **(F)** The normalized oxygen consumption was compared in wild type and two [*rps-10(0)* to wild-type] rescued strains (rescue-1, rescue-2). P values represent the results of a paired, two-tailed Student’s *t* test with Bonferroni correction comparing each rescue strain to the stage-matched wild-type control. **(G)** The relative intensity distribution of TOMM-20::TagRFP and COX-4::GFP levels in *rps-10 (0)/+* mutants and stage-matched wild-type controls are shown. Fluorescence intensities *rps-10 (0)/+* mutant animals were normalized to the median of stage matched wild-type intensities. P values represent a two-tailed Student’s *t* test. Data distribution was assumed to be normal but this was not formally tested. The bar represents interquartile distribution and the bold horizontal line represents the median. **(H)** Normalized oxygen consumption rates were plotted for *gcn-1(n4827)* mutants and *gcn-1* RNAi compared to their controls (wild type and non-target RNAi) (left). The effects of *rps-10* and *rps-23* RNAi in a wild-type background (middle), alongside the *rps-10* and *gas-1* RNAi effects in *gcn-1(n4827)* mutants (right), with respective controls for each group are shown. Each point represents the ratio of gene-specific RNAi to the respective non-target RNAi for each biological replicate. The bold line indicates the median. P values represent the results of a paired, two-tailed Student’s *t* test with Bonferroni correction, comparing each gene-specific RNAi to the non-target RNAi control. Data distribution was assumed to be normal but this was not formally tested. All experiments were performed in at least three biological replicates, and the animals were grown at 16°C. Superscript numbers denote the specific balancers compared between an RP mutant and its wild type counterpart. Balancer chromosomes are denoted as follows: +^1^ = *tmC20*, +^2^ = *tmC5*, +^3^ = *mIn1*, +^4^ = *nT1*. “*n*” at the top of each graph represents the total number of animals when all biological replicates combined. For comparisons, roughly equal number of animals were used.

The *rps-10(0)/+* mutants exhibited significant decreases in mitochondrial membrane potential, as indicated by significant MitoTracker accumulation (P < 0.001, independent Student’s *t* test) and elevated ADP/ATP ratios (P = 0.003, paired Student’s *t* test), compared with stage-matched controls (Fig. 5, A and B). These results reveal that the *rps-10(0)/+* mutants display a significant disruption in cellular energy homeostasis in addition to compromised mitochondrial membrane potential.

Having observed disruptions in mitochondrial function in *rps-10(0)/+* but not large subunit RP mutants, we assayed oxygen consumption rates in small subunit RP mutants. *rps-10(0)/+* mutants showed a reduction in oxygen consumption in comparison to stage-matched controls (P < 0.05, paired Student’s *t* test), a trend not observed in *rps-23(0)/+* (P > 0.1, paired Student’s *t* test) (Fig. 5 C). To ensure decreased oxygen consumption is not due to smaller body size, we quantified the body length and width of *rps-10(0)/+* animals, relative to controls, and found no significant difference between them (Fig. S4 D, width P = 0.48, length P = 0.080).

Given that energy and mitochondrial functionality changes were specific to the *rps-10(0)/+* mutants, we sought to determine if they were caused by a background mutation introduced during the CRISPR-mediated early stop integration or possibly due to the maternal inheritance of defective mitochondria. To address these possibilities, *rps-10(0)/+* hermaphrodites were used to reintroduce the wild-type *rps-10* sequence, leveraging the unique SuperPAM (GGNGG) sequence inserted during the creation of the mutation (Fig. 5 D). This procedure yielded two independent wild-type rescue strains from the F1 generation, each carrying two copies of the reverted wild-type *rps-10* gene. These strains displayed MitoTracker intensities and oxygen consumption rates comparable with control groups, suggesting the observed mitochondrial defects are specific to the single-copy loss of the *rps-10* gene and not related to mitochondrial biogenesis or maternal inheritance (Fig. 5, E and F, P > 0.4, independent and paired Student’s *t* test, respectively).

Additionally, we investigated whether mitochondrial defects observed in *rps-10(0)/+* mutants stemmed from changes in mitochondrial biogenesis or overall abundance. Specifically, we measured mitochondrial DNA levels and observed no differences in mitochondrial DNA content when compared with stage-matched wild-type controls (Fig. S4 E). Furthermore, we integrated fluorescent reporters for outer and inner mitochondrial membrane components (TOMM-20::Tag-RFP and COX-4::GFP [Raiders et al., 2018]) into *rps-10(0)/+* mutants and detected no significant differences in fluorescence intensity compared with stage-matched controls (Fig. 5 G, P > 0.3, independent Student’s *t* test). Taken together, these results indicate that the observed mitochondrial respiration deficits in *rps-10(0)/+* animals are due to defects in mitochondrial functionality rather than decreased abundance.

Considering the established connections between eIF2α, the integrated stress response (ISR), and mitochondrial function (Baker et al., 2012; Raini et al., 2017; Back, 2020), we investigated the role of GCN1, a key factor in the ISR that activates eIF2α kinase GCN2 (Garcia-Barrio et al., 2000; Hinnebusch 2005). GCN1’s activation of GCN2 requires its interaction with ribosomes (Marton et al., 1997; Sattlegger and Hinnebusch, 2005) and a cryo-EM study has elucidated that GCN1 binds to collided ribosomes at the interface of the small and large subunits for its role in ribosome quality control (RQC) (Pochopien et al., 2021). Although this structure does not show a physical interaction between RPS10 and GCN1, such a link was implicated through yeast two-hybrid interactions, and a correlation between decreased levels of RPS10 and reduced eIF2α phosphorylation, implying a compromised activation of GCN2 (Lee et al., 2015). Decreasing *gcn-1* expression, either through RNAi or a loss-of-function mutation (*n4857*) (Hirose and Horvitz, 2014), resulted in modestly lower oxygen consumption rates (∼30% and 20% reduction, respectively, with P values 0.12 and 0.06, paired Student’s *t* test) (Fig. 5 H, left plot). RNAi-mediated knockdown of *rps-10* resulted in approximately a 50% reduction in oxygen consumption in a wild-type background (Fig. 5 H, middle plot, P = 0.097). However, in *gcn-1(n4857)* mutants, *rps-10 RNAi* did not exacerbate the decrease in oxygen consumption (Fig. 5 H, right plot, P = 0.5, paired Student’s *t* test), despite causing developmental delays, thereby validating the effective knockdown of *rps-10*. As a control, we used RNAi against *gas-1*, a gene encoding an ETC component that could lower oxygen consumption even more in the *gcn-1(n4857)* mutants suggesting a floor level of oxygen consumption was not obtained in *gcn-1(n4857)* mutants (Fig. 5 H, right plot, P = 0.02, paired Student’s *t* test). These results overall suggest that (1) disruption in *gcn-1* could lead to reduced mitochondrial function, (2) in the absence of functional GCN-1, RPS10’s effect on mitochondrial function might be minimized or that a compensatory mechanism is activated.

### Conserved response to ribosomal protein haploinsufficiency

**Figure S5.**
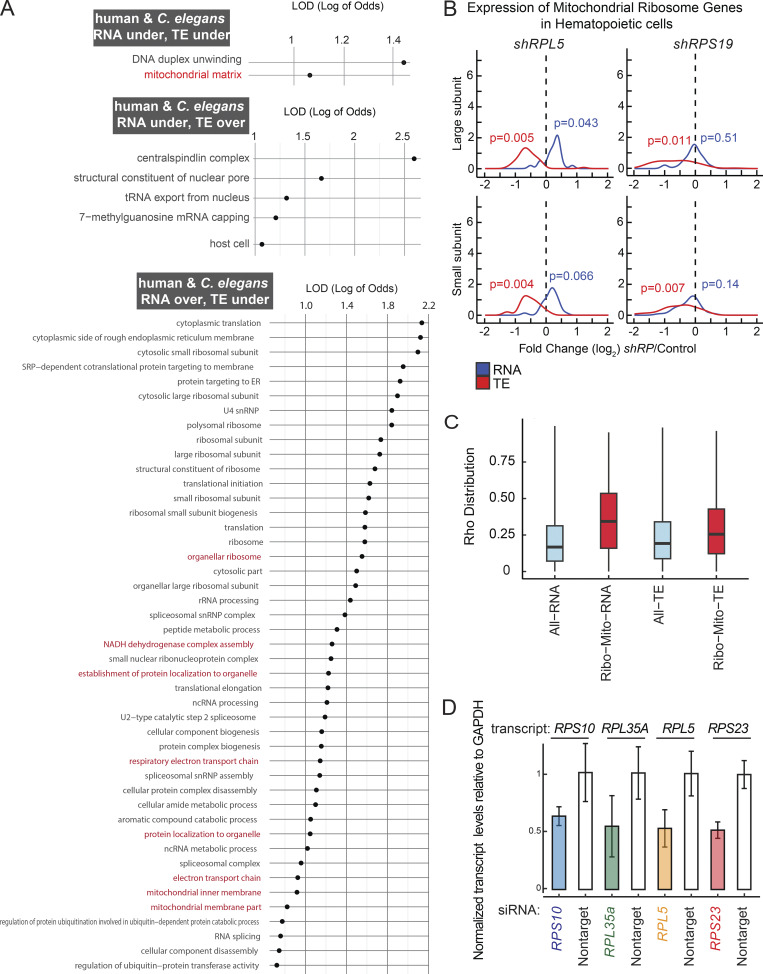
**Enriched GO categories indicative of translational control in *C. elegans* and humans, quantification of ribosomal protein gene expression in K562 cells and relative ADP/ATP ratios in response to RP siRNA treatments. (A)** Log_2_ enrichment (LOD - log of odds ratio) values are plotted for significant gene ontology (GO) categories with LOD >1 and containing fewer than 300 genes (P values <0.05). The plot displays enriched GO categories that display unidirectional or bidirectional regulation at RNA and TE level in both *C. elegans rpl-5(0)/+* mutants and in sh*RPL5* knockdown in human hematopoietic progenitor cells (left). All GO enrichment lists are provided in Table S7. Human progenitor data was reanalyzed and retrieved from Khajuria et al. (2018). GO enrichment analysis were performed using Funcassociate 3.0 (Berriz et al., 2009). **(B)** Log_2_ RNA expression and translation efficiency differences were plotted along the y-axis for all mitochondrial ribosomal protein genes that belong to large subunit (top) and small subunit (bottom) were plotted for *shRPL5* and *shRPS19* knockdown in hematopoietic cells (Khajuria et al., 2018). For statistical analysis, ROAST multivariate gene expression analysis was conducted (Wu et al., 2010). **(C)** The distribution of absolute correlations among a total of 51,465,585 gene pairs across the entire dataset, in contrast with the specific subset of 21,905 pairs involving ribosomal (ribo) and mitochondrial (mito) genes was plotted. Notably, the ribo–mito gene pairs are exclusive combinations of ribosomal genes correlated with mitochondrial genes and vice versa, without including any ribosomal-to-ribosomal or mitochondrial-to-mitochondrial correlations. This plot provides an unrestricted view of the correlation distribution, displaying the entire spectrum of correlations without applying a predefined cutoff (unlike Fig. 7 A), to fully encapsulate the breadth of gene interactions within the dataset. **(D)** Normalized transcript levels of target ribosomal protein genes (*RPS10*, *RPL35A*, *RPL5*, and *RPS23*) relative to GAPDH were quantified following siRNA treatment in K562 cells. The results were plotted to illustrate the changes in expression for each ribosomal protein gene used in mitotracker intensity measurements shown in Fig. 7 C. Data was driven from three biological replicates, error bars represent standard error.

The similarity of symptoms between DBA and Pearson syndrome (Gagne et al., 2014), the role of mitochondria in hematopoiesis (Fontenay et al., 2006), and the observed lack of coordination in the expression of mitochondrial components in DBA patients (Panici et al., 2021) led us to re-examine the RNA expression and TE of genes upon knockdown of the two most frequently mutated RPs (sh*RPS19* and sh*RPL5*) in DBA patients using hematopoietic cells (Khajuria et al., 2018). Specifically, we investigated the conserved orthologs and expression differences in *rpl-5(0)/+ C. elegans* mutants and RPL5 knockdown in human hematopoietic cells (Table S7). We particularly wondered if there is conserved unidirectional or bidirectional regulation at the RNA and TE levels impacted by reduced levels of *RPL5* across *C. elegans* and human cells. Similar to our previous results, which suggested translational control to maintain ribosome numbers (Fig. 3 B and Fig. S3 D), we observed significant functional GO term enrichments for both cytoplasmic large and small ribosomal subunits (6.5- and 5.3-fold enrichment, respectively, with P values <0.01), characterized by increased RNA levels while TE was decreased when data from *C. elegans* and humans were combined (Fig. S5 A and Table S7).

**Figure 6.**
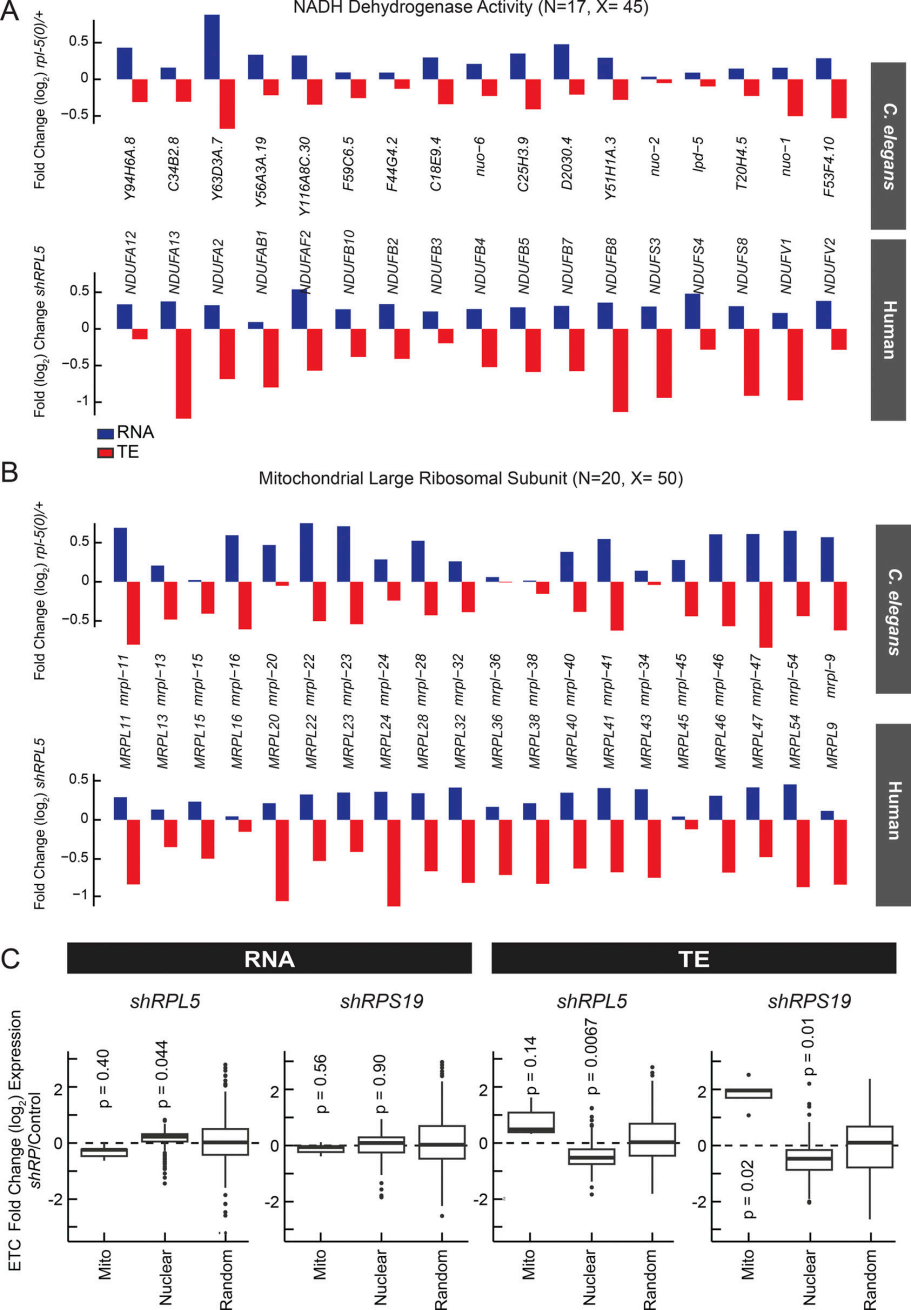
**Conserved translational regulation in *rpl-5(0)/+* animals and *shRPL5* knockdown in hematopoietic cells. (A and B)** Functional gene ontology (GO) enrichment revealed that 17 out of 45 genes within complex I of electron transport chain (the NADH dehydrogenase activity) (A) and 20 out of 50 genes associated with the mitochondrial ribosome large subunit (B) exhibit similar expression patterns (RNA over-, TE underexpressed) in both *C. elegans rpl-5(0)/+* mutants and *shRPL5* knockdown in hematopoietic progenitor cells (P_adj_ < 0.05, Funcassociate 3.0). Y axis represents log_2_ fold change estimates for RNA (blue bars) and TE (red bars) levels, separated by species. Each gene name is provided at the bottom part of the graph. Human and *C. elegans* gene orthologs are aligned. **(C)** RNA expression and TE differences were plotted along the y-axis for mitochondrial-encoded (mito) or nuclear-encoded (nuclear) electron transport chain (ETC) genes, as well as for randomly selected genes, for *RPL5* and *RPS19* knockdown in blood progenitor cells (Khajuria et al., 2018). For statistical analysis of RNA level or TE level changes (RP/control) in mitochondrial-encoded (mito) or nuclear-encoded (nuclear) ETC genes, ROAST multivariate gene expression analysis was conducted (Wu et al., 2010). P values are provided on each box plot.

Among unidirectional GO enrichments, we observed approximately twofold enrichment in the categories of DNA unwinding and mitochondrial matrix among genes that were underexpressed both at the RNA and TE level in *C. elegans* and human cells in response to *RPL5* reduction (Fig. S5 A and Table S7). Furthermore, distinct GO categories related to mitochondrial components, especially those associated with the ETC and mitochondrial ribosomes, showed an increase in RNA levels coupled with a decrease in TE (Fig. S5 A). This pattern is consistent with our results in *C. elegans*, suggesting that the effects on the ETC are indirect, and primarily due to impaired mitochondrial translation. Particularly, significant enrichment of the categories of complex I of the ETC (NADH dehydrogenase activity, Fig. 6 A) and the large subunit of mitochondrial ribosomes (both categories are 2.8-fold enriched, P values <0.01, Fig. 6 B) indicate a potentially conserved translational buffering mechanism for mitochondrial ribosomes and ETC components in the face of reductions in cytoplasmic ribosomal machinery.

While we observed significantly reduced TE for nuclear ETC components, and both subunits of the mitochondrial ribosome following reductions in *RPS19* and *RPL5* (P < 0.05, ROAST, Fig. 6 C and Fig. S5 B), mitochondrially encoded ETC components were increased at the TE level (Fig. 6 C, P = 0.14, P = 0.02, for *RPL5* and *RPS19* reduction respectively, ROAST). These findings overall highlight a broadly consistent effect of *RPL5* reduction on the RNA and TE of critical mitochondrial components in both *C. elegans* and humans, pointing toward a conserved regulatory mechanism.

### Expression coordination between ribosomal and mitochondrial components in human cells and the impact of RPS10 reduction on mitochondrial activity

**Figure 7.**
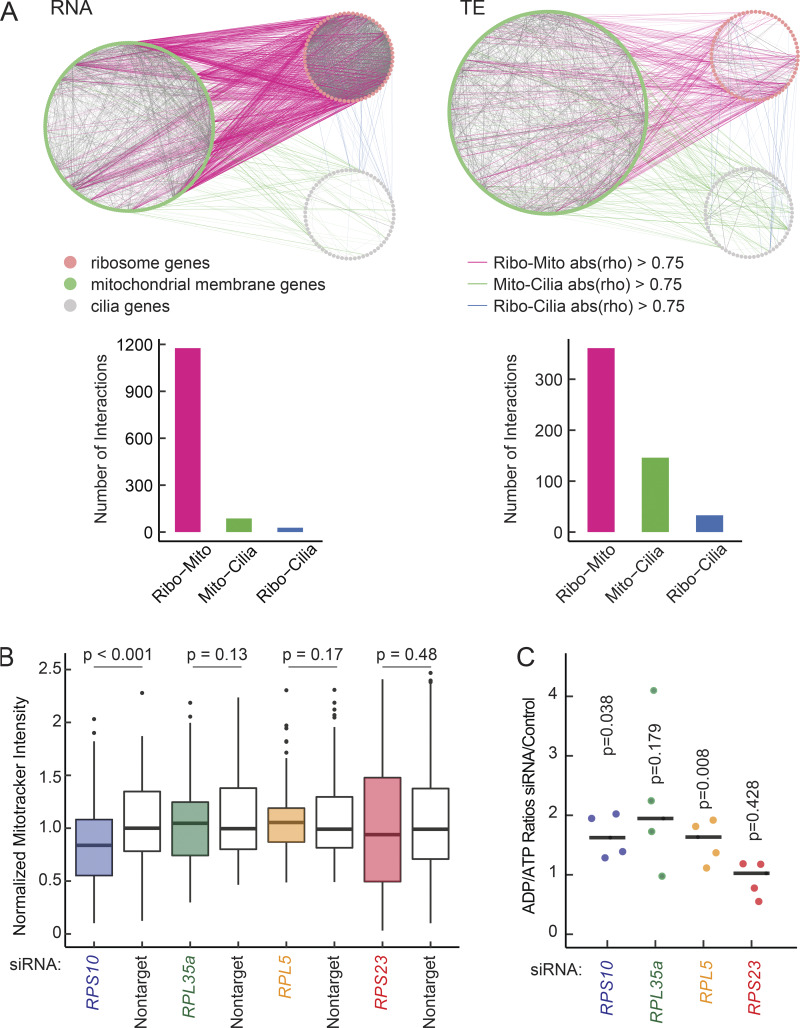
**Tight coordination between ribosomal and mitochondrial components in human cells and implications of RPS10 reduction on mitochondrial activity. (A)** Proportionality across all genes was calculated based on 13 human cell lines derived from different individuals (Cenik et al., 2015). Rho represents a measure of proportionality, indicating how changes in one gene’s expression are proportionate to changes in another. The significant proportional relationships (rho >0.75) between ribosomal and mitochondrial membrane genes were identified both at the RNA level (top left) and at the translation efficiency level (TE) (bottom left). These interactions were visualized by line interaction maps and compared to an unrelated dataset (cilia genes) to assess specificity. The number of interactions is displayed at the bottom for RNA and translation efficiency values. Ribosome genes are shown in pink, mitochondrial membrane genes are in green and cilia genes are colored in gray. **(B)** Normalized MitoTracker intensities in response to ∼50% reduction of *RPS10*, *RPL35A*, *RPL5*, and *RPS23* transcripts were plotted in comparison to nontarget siRNA. Each gene specific RNAi data was normalized to the median of non-target RNAi control. MitoTracker intensity was quantified for at least 100 cells in three biological replicates, and RNA levels were quantified by quantitative PCR for each replicate (Fig. S5 D). To evaluate significant differences between RP RNAi and wild-type controls, P values were calculated using a two-tailed Student’s *t* test. Data distribution was assumed to be normal but this was not formally tested. **(C)** ADP/ATP ratios in response to ∼50% knockdown of *RPS10*, *RPL35A*, *RPL5*, and *RPS23* transcripts in comparison to non-target siRNA are shown. Each point represents the ADP/ATP ratio of a specific RP siRNA compared to non-target siRNA for each biological replicate. The bold line represents the median of the biological replicates. To assess differences in the ADP/ATP ratio between each RP RNAi and non-target RNAi, a paired two-tailed Student’s *t* test was performed. P values are displayed above each sample. Data distribution was assumed to be normal but this was not formally tested.

To elucidate the gene expression regulatory mechanisms linking mitochondria and ribosomes in human cells, we performed an unbiased co-expression analysis at the transcription and translation levels across lymphoblastoid cells derived from 13 individuals. Specifically, we quantified the similarity of expression patterns across all genes using a compositional proportionality metric (Quinn et al., 2017; Quinn et al., 2019). This comprehensive analysis unveiled a significant correlation between ribosomal and mitochondrial membrane genes, evidenced by over 1,000 significant interactions (Fig. 7 A and Table S8). These findings suggest a highly coordinated regulation of ribosomal and mitochondrial gene expression in human cells (Fig. S5 C), highlighting the interplay between these essential cellular components.

Building upon these insights into the coordinated expression of ribosomal and mitochondrial genes, we investigated how reductions in cytoplasmic RPs affect mitochondrial function, a subject that has been relatively unexplored in human cells. We used K562 leukemia cell line to examine the impacts of reduced levels of specific RP transcripts, *RPS10*, *RPL35A* (the ortholog of *C. elegans rpl-33*), *RPL5*, and *RPS23*. Using siRNA knockdowns, we achieved a ∼50% reduction in their transcript levels (Fig. S5 D). The mitochondrial membrane potential, assessed using MitoTracker, demonstrated significant decreases in activity following *RPS10* reduction (Fig. 7 B, P = 5e-4, independent Student’s *t* test), accompanied by an increase in the ADP/ATP ratios (Fig. 7 C, P < 0.05 for *RPS10* siRNA, paired Student’s *t* test), highlighting the critical role of RPs in supporting mitochondrial energy metabolism.

These observations collectively emphasize a balance between ribosomal and mitochondrial gene expression, which is crucial for cellular energy production and metabolic health. The differential regulation of TE for mitochondrially versus nuclear-encoded ETC components suggests adaptability to counteract the effects of RP reduction, with a substantial impact of *RPS10* reduction on mitochondrial activity and energy metabolism.

## Discussion

Here, we investigated the effects of RP haploinsufficiency within the *C. elegans* model, focusing on its cellular and developmental consequences. Previous studies have established that functional deficiencies in RPs can trigger significant developmental and physiological alterations across a range of model organisms, including *Drosophila* and yeast (Marygold et al., 2007; Marygold et al., 2005; Cheng et al., 2019; Maitra et al., 2020; Wilkes et al., 2020; Morgado-Palacin et al., 2015; Horos et al., 2012). In addition to yeast, which possesses widespread genomic duplications of RP genes (Wolfe and Shields, 1997), or *Drosophila*, which also utilizes specific pathways including the Xrp1-Dil8 system activated in response to RP knockdown (Colombani et al., 2012; Boulan et al., 2019), our nematode model provides a unique perspective on the consequences of single-copy loss for four RP genes (*rps-10*, *rps-23*, *rpl-5*, and *rpl-33*).

In this study, we uncovered insights into the relationship between ribosome production, mitochondrial integrity, and cellular metabolism, highlighting the roles of RPs beyond their traditional role in protein synthesis. We found that mitochondrial and nuclear-encoded components of the ETC are differentially regulated in response to RP reduction or haploinsufficiency in humans and *C*.* elegans*, respectively. This differential regulation suggests a conserved strategy to maintain mitochondrial function despite variations in cytoplasmic ribosome component expression, indicating a fundamental connection between ribosomal assembly and metabolic regulation.

Further supporting the tight coupling between ribosomal and mitochondrial components, we discovered significant covariation of these transcripts at both the RNA and TE levels. At the translation level, this link may be mediated by factors such as TRAP1, which is associated with both mitochondrial and cytoplasmic ribosomes and controls the translation elongation rate (Avolio et al., 2023). Furthermore, our observations of mitochondrial morphology differences across all RP haploinsufficient mutants, especially those related to RPS10 reduction, indicate substantial impacts on mitochondrial activity in both species. This alteration affects cellular energy homeostasis and suggests that buffering mechanisms for the maintenance of mitochondrial health in response to environmental inputs may be compromised. Although all RP haploinsufficient mutants exhibit reduced TE of mitochondrially encoded ETC components, only the *rps-10(0)/+* mutant shows significant functional defects in mitochondrial activity and energy levels. Given that the reduction in TE of mitochondrial ETC components in *rps-10(0)/+* is not greater than that observed in the other mutants, this suggests that RPS-10 may play a unique role in ribosome assembly or function that more profoundly affects mitochondrial function compared with other RPs. Alternatively, other RP haploinsufficient mutants might activate downstream compensatory pathways that balance the protein levels of ETC components to preserve mitochondrial function, whereas the *rps-10(0)/+* mutant may be deficient in activating these compensatory mechanisms, leading to the observed functional defects. Finally, it is possible that RPS-10 interacts with specific mitochondrial factors or signaling pathways critical for maintaining mitochondrial integrity and function, and its haploinsufficiency disrupts these interactions more significantly than the haploinsufficiency of other RPs.

Our results also suggest that disruption of *gcn-1* leads to reduced mitochondrial function, and that in the absence of functional GCN-1, the effect of RPS-10 on mitochondrial function is minimized or a compensatory mechanism is activated. The lack of additional mitochondrial dysfunction upon RPS-10 reduction in the *gcn-1* mutant background indicates that GCN-1 is required for RPS-10’s impact on mitochondrial function, suggesting a potential regulatory pathway in which GCN-1 modulates the effects of RPS-10 on mitochondria.

In human leukemia cells, we also observe a significant reduction in mitochondrial activity and overall energy levels only when *RPS10* transcripts are reduced by 50%. This finding implies that the unique effect of RPS-10 may be a conserved phenomenon across species. Interestingly, although the mitochondrially-encoded ETC components were differentially translated, their levels significantly increased in previously published *shRPL5* and *shRPS19* datasets. This suggests that compensatory translation between mitochondrial and nuclear-encoded components of the ETC is important for maintaining homeostasis and that such compensation might be insufficient in the case of *RPS10* reduction.

The mitochondrial metabolism disruptions in *rps-10* haploinsufficient mutants mirror metabolic changes upon loss of *Rpl22A* in yeast, such as the decreased one-carbon metabolism, a pathway within mitochondria (Maitra et al., 2020). Furthermore, the assembly of RPS10 in *S. cerevisiae* is disrupted when there is a deficiency of Ltv1, a biogenesis factor. While Ltv1 deficiency provides a growth advantage in some conditions, it also predisposes cells to oxidative stress (Collins et al., 2018). This raises the question of whether the reduction of RPS10 in human leukemia cells and *C. elegans*, leading to mitochondrial dysfunction, is tied to the similar effects seen with reduced Ltv1, given the absence of Ltv1 leads to the absence of RPS10 similar to the effect conferred by the mutation or knockdown.

In addition, previous studies have implicated distinct roles of RP paralogs on mitochondrial activity. Specifically, in *S*.* cerevisiae*, only one of the two paralogs of RPL1, RPL2, and RP26 display defective mitochondrial morphology, and RPL1b is involved in the translation of respiration-related genes (Segev and Gerst, 2017). Similarly, in mice, the paralog of RPL3, RPL3L, is exclusively expressed in skeletal muscle and heart tissues, and its lack is associated with altered ATP levels. Interestingly, RPL3L-containing ribosomes interact with mitochondria and potentially interfere with mitochondrial function (Milenkovic et al., 2023).

The impact of RP haploinsufficiency on mitochondrial TE also offers insights into the adaptive mechanisms in response to ribosomal dysfunction. For instance, the overexpression of glutathione transferases observed in *C. elegans* mutants suggests an increased cellular reliance on antioxidant defenses, likely a compensatory response to elevated ROS levels due to mitochondrial dysfunction (Kapralova et al., 2020).

This adaptation reflects an evolutionarily conserved strategy to safeguard cellular functionality under genetic stress, further emphasizing the interdependence between mitochondrial integrity, RP function, and cellular stress responses. Additionally, the regulatory mechanism whereby mitochondrial dysfunction downregulates cytoplasmic protein translation through the phosphorylation of eIF2α by stress-related kinases highlights the reciprocal relationship between mitochondrial stress and cytosolic translation (Guo et al., 2020; Baker et al., 2012; Fessler et al., 2020). By elucidating the compensatory mechanisms that preserve mitochondrial function in the face of RP loss, we contribute to a deeper understanding of cellular resilience.

## Materials and methods

### *C. elegans* maintenance and experimental conditions

*C. elegans* strains used in this study were sourced from the *Caenorhabditis* Genetics Center (CGC), supported by the NIH Office of Research Infrastructure Programs (P40 OD010440). Standard cultivation practices involved growing the nematodes on Nematode Growth Medium (NGM) plates, which were seeded with the *E. coli* OP50 strain. To ensure genetic stability, particularly to minimize recombination and prevent the loss of balancer chromosomes, we incubated the animals at 16°C, with the exception of experiments investigating mitochondrial morphology under stress (see Fig. 4, B and C). In these experiments, we did not initially notice differences in the mitochondrial structures of young adult worms maintained at 16°C. To further explore mitochondrial dynamics under stress, we allowed the animals to mature into 3-day-old adults at 16°C, then transferred adults to a stress-inducing environment at 23°C for 2 days prior to the imaging procedures.

Balancer chromosomes are denoted as follows: +^1^ = *tmC20*, +^2^ = *tmC5*, +^3^ = *mIn1*, +^4^ = *nT1*. Balancer chromosomes were used in all control strains, except for the analysis of mitochondrial morphology (Fig. 4, B and C). For the *C. elegans* experiments depicted in Figs. 1, 2, 3, 4, and 6, the following balancers were used for each haploinsufficient RP strain, along with corresponding wild type/balancer controls: *rps-10(0)/+*^*1*^, *rps-23(0)/+*^*2*^, *rpl-5(0)/+*^*3*^, and *rpl-33(0)/+*^*3*^. For Fig. 5 C, *rps-23(0)/+*^*4*^ was used as +^*3*^ (*nT1*) is homozygous lethal and facilitates the collection of a large sample of animals.

### Strain crossing procedures

#### ESC299 *(rpl-5[cc5998,A166X]/mIn1, skn-1(zj15) IV)*

Phase 1: Crossed male wild type/*mIn1* (non-dumpy, GFP+) with QV225 (*skn-1(zj15)*) to produce F1 hermaphrodites (wild type/*mIn1*, *skn-1(zj15)*/wild type) with GFP. Dumpy F2 progeny were isolated and genotyped for *skn-1(zj15)* homozygosity. Phase 2: Non-dumpy, non-GFP male *rpl-5(0)/+* were crossed with dumpy, GFP+ hermaphrodites homozygous for *mIn1* and *skn-1(zj15)* from Phase 1. F2 progeny were genotyped for *skn-1(zj15)* homozygosity and *rpl-5(0)* heterozygosity, ensuring all displayed pharyngeal GFP to avoid *rpl-5(0)* homozygous developmental arrest.

#### ESC733 *(rps-10[cc2557,T8X], cox-4(zu476[cox-4::eGFP::3XFLAG])/tmC20)*

Phase 1: Crossed male wild type*/tmC20* (non-dumpy, mVenus positive) with JJ2586 (*cox-4(zu476[cox-4::eGFP::3XFLAG])*) to select F1 hermaphrodites displaying both body GFP and pharyngeal mVenus. After three generations, dumpy F2 progeny were made homozygous for body *cox-4::eGFP::3XFLAG* signal. Phase 2: Non-dumpy, non-GFP *rps-10(0)*/wild type males were crossed with GFP positive, dumpy hermaphrodites homozygous for *tmC20* and *cox-4(zu476[cox-4::eGFP::3XFLAG])* from Phase 1. F2 progeny were visually genotyped for *COX-4::eGFP* homozygosity and *rps-10(0)* heterozygosity.

#### ESC613 *(rps-10[cc2557,T8X]/tmC20, tomm-20::Tag-RFP V)*

Phase 1: Crossed male wild type/*tmC20* (non-dumpy, mVenus positive) with ESC158 (*tomm-20::Tag-RFP*) to collect F1 hermaphrodites displaying both body RFP and pharyngeal mVenus signal. Dumpy F2 progeny were isolated and confirmed for *tomm-20::Tag-RFP* homozygosity via positive body RFP signal. Phase 2: Non-dumpy, non-GFP *rps-10(0)/tmC20* males were crossed with dumpy, RFP positive hermaphrodites homozygous for *tmC20* and *tomm-20::Tag-RFP* from Phase 1. F2 progeny were visually genotyped for *tomm-20::Tag-RFP* homozygosity and *rps-10(0)* heterozygosity.

All strains used in this study are provided in Table S9.

### Strain generation via CRISPR-Cas9

Strains ESC614 and ESC615 were derived from heterozygous animals carrying the genotype *rps-10[cc2557,T8X]/tmC20, [unc-14(tmIs1219) dpy-5(tm9715)] I*. Young adult heterozygotes were injected with a CRISPR injection mix, which included a 2.5 µM homologous recombination template (ESC-AS-130), 50 ng/μl guide RNA plasmid (pAS14), and 50 ng/μl Cas9-expressing plasmid (pDD132), adapting the co-conversion method (Arribere et al., 2014). Rescue mutations were initially selected by identifying balancer chromosome, *tmC20*-free adult animals, characterized by the absence of pharyngeal GFP markers and the avoidance of both the developmental arrest associated with homozygous *rps-10(0)* and the uncoordinated phenotype of *tmC20, [unc-14(tmIs1219) dpy-5(tm9715)]* progeny. These potential rescue mutations were subsequently confirmed through PCR amplification and Sanger sequencing.

We used CRISPR-Cas9 to generate a strain expressing TOMM-20::TagRFP in an otherwise N2 background. We annealed and inserted sense and anti-sense guide DNA oligos (ESC-RR-21 and ESC-RR-22) into pRB1017, a vector that expresses guide RNA with U6 promoter. Cas9 protein was expressed from eft-3 promoter using the plasmid pDD162. The 5′ and 3′ homology arms were amplified ∼500 bp upstream of *tomm-20* stop codon using oligos ESC-RR-17 and ESC-RR-18, and ∼500 bp downstream of stop codon using ESC-RR-19 and ESC-RR-20. *C. elegans* genomic DNA was used for these amplifications. These homologous arms were used to replace the ccdB in pDD284(TagRFP-T^SEC^3xFlag). All plasmids for microinjection were purified using the PureLink HiPure Plasmid Miniprep Kit (#K210002; Invitrogen). Oligo sequences used to generate these plasmids are provided below as well as in Table S9. The downstream injection and selection process was done exactly as explained in the supplementary methods section utilizing a self-excising hygromycin selection-based CRISPR-Cas9 protocol (Dickinson et al., 2015).

All strains and DNA sequences that were generated or used in this study are provided below in the methods section as well as in Table S9.

### Proteomics analysis

*C. elegans* animals were bleach-synchronized (adult animals were incubated with 0.5 M NaOH and 1% NaClO for 6 min to extract eggs) and subsequently grown on NGM plates until they reached the L4 stage. At this point, the animals were collected using 50 mM NaCl. To ensure the removal of bacteria and prepare for proteomic analysis, the collected animals underwent a series of serial centrifugations at 300 × *g* for 10 min with 3% sucrose in 50 mM NaCl. The animals were then resuspended in Laemmli Buffer (1610737; Bio-Rad), supplemented with PMSF (36978; Thermo Fisher Scientific) and BME (11411446001; Sigma-Aldrich), and immediately flash-frozen. Next, the samples were subjected to mechanical disruption via manual bead-beating to ensure thorough digestion of the protein content. The resulting protein fractions were then loaded onto NuPAGE Bis-Tris Gels (4–12%) (NP0335BOX; Thermo Fisher Scientific) and ran using MES SDS Running Buffer (B0002; Thermo Fisher Scientific). The gel was briefly stained by Coomassie staining (#1610786; Bio-Rad). Post-electrophoresis, the gels within the top stacking portion were cut into sections using a razor for further processing. The excised gel sections underwent trypsin digestion before the peptides were desalted. These prepared samples were then analyzed using a Dionex Liquid Chromatography system coupled with an Orbitrap Fusion 2 mass spectrometer. The analytical run was conducted over a 120-min period to ensure comprehensive peptide identification and quantification. Protein identification was provided by the UT Austin Center for Biomedical Research Support Biological Mass Spectrometry Facility (RRID:SCR_021728).

For data analysis, raw outputs, including label-free quantification (LFQ) values and peptide counts, were processed using Proteome Discoverer version 2.5 (Orsburn, 2021). This software facilitated the mapping of the data against the *C. elegans* reference database. Further quantitative analysis was performed utilizing the DEP (Differential Expression Proteomics) package in R, accessible via the Bioconductor project (https://rdrr.io/bioc/DEP/man/DEP.html). Proteomics analysis by DEP is provided (Table S1).

### Body area measurement

For the body area and length assays, animals were synchronized through a 2-h egg-laying period and subsequently grown until L4 development. Prior to imaging, animals were anesthetized at room temperature using 10 mM Levamisole. The animals were imaged using a Leica Stellaris 8 confocal microscope with a Leica K5 microscope camera. Leica Application Suite X was used for image acquisition. Images were acquired through differential interference contrast (DIC) with a 20× (NA 0.4) objective. ImageJ software was used to measure the body area by drawing segmented lines along the length of each animal from head to tail (Fig. 1 D). Body length was quantified by drawing segmented lines from head to tail, and body width was quantified by drawing segmented lines across the midsection of the body (Fig. S4 D). Body area (Fig. 1 D) and body length and width (Fig. S4 D) were measured using ImageJ software. All measurements were normalized using the median body area of the respective wild type/balancer control group. Statistical differences between the average normalized body area of mutant and control animals were measured using a two-tailed Welch’s two-sample *t* test.

### Brood size determination

Animals were synchronized using a 2-h egg-laying window. Heterozygous animals, either carrying an RP gene mutation and a balancer chromosome or a wild-type gene with the corresponding balancer, were individually transferred to fresh NGM plates. A single animal was moved to a new plate roughly every 24 h. Hatched progeny from each animal were counted (Fig. 1 E and Fig. S1 B). Differences in the mean brood size of each mutant, compared to its respective control, were quantified using a two-tailed Student’s *t* test.

### Lifespan assay

The lifespan assay was conducted on solid Nematode Growth Media, both with and without the addition of FUDR (Van Raamsdonk and Hekimi, 2011; Sutphin and Kaeberlein, 2009). Following synchronization via egg-laying, animals were placed on NGM plates with or without 0.5 µM FUDR (Van Raamsdonk and Hekimi, 2011; Sutphin and Kaeberlein, 2009) (Fig. S1 C and Fig. 1 F, respectively). The animals were grown at 16°C, and the survival of each nematode was assessed every 24 h. Animals on the NGM plates without FUDR were transferred every 2 days. For FUDR plates, concentrated OP50 spun at 2,000 rpm was used as a food source during the lifespan assay. Statistical analysis was conducted using the Log-rank test with Bonferroni correction for multiple comparisons (Yang et al., 2011).

### Puromycin labeling and western blotting

Puromycin pulse labeling was performed on three replicates. Animals were bleach-synchronized and collected at the L4 stage. Plates with L4 worms were supplemented with OP50 and 0.5 mg/ml puromycin dihydrochloride from *Streptomyces alboniger* (P8833; Sigma-Aldrich). These worms were incubated at 20°C and pulse-labeled for 4 h (Weaver et al., 2020). After labeling, worms were washed twice with 5% sucrose and twice with 50 mM NaCl before being flash-frozen with 5 μl glass beads.

For worm lysis, Laemmli buffer supplemented with PMSF and β-mercaptoethanol (BME) was added to each sample before bead beating for 30 s. Samples were spun down through centrifugation for 2 min at 15,000 × *g*. The samples were then boiled at 95°C for 5 min. An additional 2-min centrifugation was performed to clear debris prior to measuring protein concentration with Qubit Protein Assay Kit (Q33211; Thermo Fisher Scientific).

Approximately 16–17 μg of protein per sample was loaded on Invitrogen NuPAGE Bis-Tris Mini Protein Gels (4–12%, 1.0–1.5 mm, NP0335; Invitrogen) and separated at 80–120 V in 1X MES SDS running buffer. Proteins were transferred onto a PVDF membrane (88518; Thermo Fisher Scientific) at 30 V for 1 h. Membranes were blocked in 1X TBS-T containing 5% milk. Puromycin and actin were probed with primary antibodies: Anti-Puromycin Antibody, clone 12D10 (Mouse, 1:2,000, MABE343; Sigma-Aldrich) and Anti-Actin Monoclonal Antibody (clone: C4) (Mouse, 1:500, 10221-880; VWR), respectively. A secondary antibody, goat anti-mouse IgG H&L (HRP) (1:10,000, ab6789; Abcam) was used in conjunction with a chemiluminescent substrate (34579; Thermo Fisher Scientific) for visualization.

All replicates were quantified by recording the average intensity in the range of ∼20–35 kDa using ImageJ, with the quantified actin band used for normalization.

### Acute oxidative stress and heat stress assays

For the acute oxidative stress and heat assays (Fig. 2 C; and Fig. S2, A–C and E), *C. elegans* were grown at 16°C. To induce proteotoxicity, translation or TORC1 inhibition, bleached eggs were pregrown on NGM plates with the following drugs prior to the stress assay: final concentrations of 100 µM rapamycin (HY-10219; MedChemExpress), 25 nM bortezomib (HY-10227; MedChemExpress), and 25 nM cycloheximide (01810-5G; Sigma-Aldrich).

For the oxidative stress assay, L4 animals were transferred to fresh OP50-seeded NGM plates spiked with 0.2 M paraquat (AC227320050; Thermo Fisher Scientific). For heat stress, L4 animals were subjected to a temperature of 37°C to induce heat stress.

For both assays, the survival rate was evaluated at room temperature across various time points, determining mortality based on the lack of movement within 15 s after being gently prodded with a platinum pick. Statistical analysis was conducted using the Log-rank test with Bonferroni correction to compare the survival distributions of multiple groups (Yang et al., 2011).

### RNA-seq and ribo-seq library preparation and data analysis

For Ribo-seq and RNA-seq procedures, L4 staged heterozygous larvae were collected, and the remaining bacteria were cleaned up using a 5% sucrose solution with 50 mM NaCl. The animals were stored in 300 μl of lysis buffer (20 mM Tris-HCl, pH 7.4, 150 mM NaCl, and 5 mM MgCl_2_, 1 μl of 1 M DTT, 10 μl of 10% Triton-X, 100 μg/ml cycloheximide) and flash-frozen in liquid nitrogen. These samples were then stored at −80°C until further use.

The frozen animal pellets were then ground to a fine powder to break cuticles in liquid nitrogen using a mortar and pestle, and the powder was collected in a 1.5 ml tube. The powder was allowed to thaw on ice before 20 U of Turbo DNAse (AM2238; Thermo Fisher Scientific) were added. Each lysate sample was divided into two parts for RNA-seq and Ribo-seq, and 1 ml Trizol (15596026; Thermo Fisher Scientific) was added to the RNAseq aliquot. After a brief vortex and incubation on ice for 15 min, the RNA concentration was measured using a Qubit RNA BR assay. RNAse I (EN0601; Thermo Fisher Scientific) was added to each Ribo-seq sample at a ratio of 150 units per 30 µg of RNA and incubated for 30 min at room temperature. The RNAse I reaction was stopped with a final concentration of 25 mM ribonucleoside vanadyl complexes (R3380; Sigma-Aldrich). The samples were loaded onto the 34% sucrose cushion prepared in lysis buffer and spun at 70,000 rpm for 4 h at 4°C using a TLA 100.3 Rotor in an Optima Ultracentrifuge (361889; Beckman). After centrifugation, the supernatant was removed to isolate the pellet, which was then dissolved in 1 ml Trizol. The samples with Trizol added for RNA-seq and Ribo-seq were briefly vortexed. Following a 5-min room temperature incubation, 200 μl of chloroform was added, and the samples were spun at 15,000 rpm for 10 min, then the aqueous layer was transferred to a new tube. The final concentration of 50 mM 3 M NaAcetate (pH 5.5), 5 mM MgCl2, and 2 μl Glycoblue coprecipitant (AM9515; Thermo Fisher Scientific) was added along with 500 μl isopropanol, and the samples were incubated overnight at −20°C. The next day, the samples were spun at 15,000 rpm, at 4°C for 60 min and the pellets were washed with 80% ethanol. The pellets were dissolved in DEPC-treated water before the subsequent steps. The Ribo-seq samples were treated with T4 PNK (EK0031; Thermo Fisher Scientific) in T4 PNK buffer for 30 min at 37°C and run on a 15% TBE urea gel. After staining SYBR Gold (S11494; Thermo Fisher Scientific) and imaging, ribosome footprints between 26 and 34 bases were cut out for further processing with D-Plex Small RNA sequencing kit with unique molecular identifiers (UMI) (C05030001, C05030021; Diagenode) for library preparation. For RNA-seq library preparation, SMARTer Stranded RNA-Seq Kit (634837; TakaraBio) was used. Three independent biological replicates were performed. Each wild-type control included two samples that were time- and stage-matched to RP mutants to avoid any gene expression changes that are due to the observed developmental delay in RP mutants. Transcriptome mapping reads for the third replicate were not sufficient (<300K reads), therefore the third replicate was removed from further analyses.

To analyze human shRPS19 and shRPL5 knockdown experiments in hematopoietic cells, raw data was downloaded from NCBI GE (GSE89183 [Khajuria et al., 2018]).

For read mapping and further processing of the data, Riboflow nextflow pipeline was utilized (Ozadam et al., 2020) (https://github.com/ribosomeprofiling/riboflow). Before mapping the unique identifier barcodes that were added in Ribo-seq, libraries were collapsed to count the number of unique RNA molecules and adapters that were removed. Reads were mapped against the *C. elegans* transcriptome (WBCel235; Ensembl) and human transcriptome (GRCh38.p14; Gencode). The Ribo-seq and RNA-seq counts obtained from the pipeline were analyzed using the edgeR pipeline in R (Robinson et al., 2010). To investigate differences in TE, ribosome-bound RNA (Ribo) levels, and RNA expression levels across the samples, three specific contrasts were constructed. Subsequently, a quasi-likelihood F-test (glmQLFTest) was employed to assess these contrasts and adjust for multiple testing errors using a false discovery rate (FDR) approach, setting a P-value threshold of 0.05 for significance. The contrast specifically designed to evaluate TE was defined as “TE_RPmutantvsControl = (RP.Ribo - RP.RNA) − (Control.Ribo - Control.RNA).” This approach allowed for a comparison of the TE between RP mutants and their respective controls. When examining significance across all RP mutants, we categorized samples into two groups: RP mutants versus controls. However, when analyzing individual mutants, each was compared to both stage-matched and time-matched controls, within the same genetic background.

RNA-seq, ribo-seq, and TE analysis results by EdgeR are provided in Tables S2, S4, and S6.

Mitochondrial genes were extracted using Mitocarta 3.0 (Rath et al., 2021). *C. elegans* and human gene orthologs were extracted using BioMart (Smedley et al., 2015). Gene expression values of all conserved orthologs between *C. elegans* and humans are provided in Table S7.

For ROAST multivariate gene expression analysis, raw counts for both RNA and TE were normalized using the edgeR package and subsequently transformed with respect to the mean-variance trend using the voom function from the limma package (Ritchie et al., 2015). Rotation gene set tests were conducted on transformed counts for RNA and TE using the roast function (Wu et al., 2010) from limma.

### Imaging and analysis of mitochondria morphology

For mitochondrial imaging, RP mutants carrying balancers and N2 worms were crossed with male PD4251 (*ccIs4251 [myo-3p::GFP::LacZ::NLS + myo-3p::mitochondrial GFP + dpy-20(+)] I; vsm-1(ok1468) IV)* worms. From these crosses, L4 animals carrying a single copy of the *myo-3p::GFP* mitochondrial marker were recovered from the transient F1 generation for use in mitochondrial imaging and analysis. These L4 animals were transferred to 23°C where they were incubated until day 3 of adulthood.

Animals were then immobilized using 10 mM Levamisole and imaged using a Leica Stellaris 8 Confocal Microscope. Leica Application Suite X was used for acquiring images (Fig. 4 B). Images were acquired at room temperature with a 63× (NA 1.4) objective. At least nine nematodes were imaged for each mutant, spanning three biological replicates, with 5–10 cells being imaged in each nematode.

Image analysis was performed using the ilastik v1.4.0 “Pixel Classification + Object Classification” pipeline (Berg et al., 2019). Briefly, raw images were input for pixel classification to separate mitochondria and nuclei from the background based on GFP signal intensity. Nuclear-localized GFP was excluded from the remaining analyses, and only mitochondrial-localized GFP was used. The object classification portion of the pipeline was used to extract various features of each individual mitochondrial object, including convexity, defect area, and branch length. For each condition, >10,000 data points were collected. Statistical comparison of mean features, specifically convexity, mean defect area, and mean branch length, between control and mutant samples was carried out using a Student’s *t* test, performed with the “t.test” function in R (https://www.R-project.org/) to compare differences in average feature measurements across mutant and control animals. Visualization of feature distributions was performed using the “ggplot” package in R (Fig. 4 C [Villanueva and Chen 2019]). The pipeline and code used in this analysis are available at https://github.com/raqmejtru/mito_image_analysis/.

### *C. elegans* Mitotracker accumulation assay

The Mitotracker staining of *C. elegans* was adapted from the protocol (Sarasija and Norman, 2018). Following bleach-synchronization, L4 animals and OP50 bacteria were collected using M9 buffer. Samples were incubated with 1 µg/ml Mitotracker CMXRos (M7512; Thermo Fisher Scientific) for 6 h at 20°C. To remove excess dye, the nematodes were then washed twice with M9 buffer. After washing, the animals were transferred to fresh NGM plates seeded with OP50 and allowed ∼1 h for foraging, which helps in clearing any dye that might have been nonspecifically accumulated in the gut. For quantitative imaging, the stained nematodes were mounted on slides prepared with 3% agarose in M9 buffer, ensuring no anesthetics were used that could potentially interfere with the fluorescence. Imaging was performed at room temperature using a 20× (NA 0.4) objective on a Leica SPE microscope using fixed fluorescent exposure and a Leica K5 microscope camera. Leica Application Suite X was used for image acquisition. The intensity of the Mitotracker accumulation per area for each animal was quantitatively measured using ImageJ software. Staining specificity was assessed through co-localization studies using a *C. elegans* strain with a CRISPR-engineered knock-in of *cox-4* gene tagged with GFP (*cox-4::GFP*), serving as a marker for mitochondrial inner membranes. These colocalization analyses were conducted using a Leica Stellaris 8 confocal microscope with a 63× (NA 1.4) objective and Leica Application Suite X. For visualization and confirmation of the staining pattern’s specificity, we compared the images to the known localization of the COX-4::GFP signal within the mitochondria (Fig. S4 C).

### ADP/ATP measurement in *C. elegans*

Bleach-synchronized L4 animals were transferred to bacteria-free NGM plates to ensure cleanliness for the assay. A selection of 50 animals was made to specifically exclude those with homozygous balancer chromosomes, and these selected worms were placed in 50 μl of M9 buffer. The prepared samples were flash-frozen in liquid nitrogen and stored at −80°C until further analysis. The method for measuring the ADP/ATP ratio in *C. elegans* was adapted from the protocol (Palikaras et al., 2015). The ADP/ATP ratio was quantitatively measured from lysis supernatant using the Sigma-Aldrich ADP/ATP Assay Kit (MAK135-1KT; Sigma-Aldrich), following the manufacturer’s instructions. Measurements were carried out on a Glomax Luminescence Microplate Reader (Promega).

### *C. elegans* oxygen consumption measurements

To measure oxygen consumption in *C. elegans*, bleach-synchronized animals were grown on NGM plates seeded with *OP50* or *HT115 E. coli* expressing specific or non-target siRNAs at 16°C until the L4 stage. The animals were collected using a 50 mM NaCl solution and underwent a cleaning process to remove bacteria. This involved centrifugation at 800 × *g* for 1 min, repeated three times with M9 buffer to ensure thorough cleansing. After cleaning, the nematodes suspended in the M9 buffer were transferred into the sealed and precalibrated chamber of O2k-FluoRespirometer (Oroboros) for the determination of the oxygen consumption rate. The oxygen consumption rate value was then normalized based on the exact number of animals that were introduced into the measurement chamber to reflect the metabolic rate per individual animal.

### *C. elegans* fluorescence intensity measurements

To measure fluorescence intensity, L4 stage-matched animals expressing TOMM-20::TagRFP and COX-4::GFP, markers for mitochondrial localization, were imaged using a Leica SPE Fluorescence DIC microscope with a 20× (NA 0.4) objective lens and a Leica K5 microscope camera. Images were acquired using Leica Application Suite X. For quantifying the fluorescence intensity per animal, the captured images were analyzed using ImageJ software. Specifically, for the measurements of COX-4::GFP fluorescence intensity, care was taken to exclude the pharyngeal area from analysis. This precaution was necessary to avoid interference from the mVenus marker present in both the *rps-10(0)/tmC20* and wild type*/tmC20* control strains.

### Proportionality analysis of human gene expression and translational efficiency

We conducted a detailed analysis of gene expression samples of 13 human individuals from diverse genetic backgrounds. The samples were selected to have Ribo-seq paired with RNA-seq data from the study GSE65912 (Cenik et al., 2015), utilizing the RiboFlow toolkit (Ozadam et al., 2020). To refine the ribosome profiling dataset further, we applied a winsorization technique to adjust for potential PCR duplication artifacts, capping nucleotide counts at the 99.5th percentile to address over-amplified outliers. We excluded 166 human genes identified as lacking polyA tails to ensure the analysis focused on high-quality gene counts. Following this exclusion, both RNA-seq and Ribo-seq data were normalized using counts per million (CPM), selecting genes with a CPM >1 in over 70% of the samples for further analysis. This process resulted in the retention of 10,145 human genes.

TE and RNA value for each gene in each sample was calculated as explained in Ribo-seq analysis section. To assess coordinated expression and TE among genes, we used the “lr2rho” function from the “propr” R package (Quinn et al., 2017), inputting centered log ratio values of TE or RNA expression for 10,145 human genes. The resulting rho values, ranging from −1 to 1, facilitated the generation of CoTE and co-expression matrices. Gene sets were curated from the GO and KEGG pathway databases, focusing on the mitochondrial membrane, ribosomal, and cilia-associated genes. Performing overlap analysis with these gene lists identified 445 mitochondrial, 65 ribosomal, and 124 cilia-associated genes. For comparability, we selected a subset of 65 genes from the cilia-associated gene set to match the ribosomal gene count. The TE and RNA levels of these 575 genes were then analyzed, focusing on interactions where the absolute rho value exceeded 0.75. Gene interactions with a rho value exceeding 0.75 were visualized using Cytoscape version 3.9.1 (Shannon et al., 2003). All Ribo–Mito interactions that had rho values higher than 0.75 are provided (Table S9).

### siRNA knockdown in K562 cells

K562 Cells (ATCC) were maintained in RPMI (11879020; Thermo Fisher Scientific) supplemented with 10% fetal bovine serum/FBS (900-108; Gemini Bio) and glucose 2 g/liter (A2494001; Thermo Fisher Scientific) and incubated at 37°C and 5% CO_2_. K562 cells were cultured until reaching 80% confluency. The cell count was assessed using a hemocytometer and Trypan Blue (15-250-061; Thermo Fisher Scientific) staining. The cells were centrifuged at 600 × *g* for 5 min, after which the supernatant was replaced with Opti-MEM (31985070; Thermo Fisher Scientific) to adjust the cell count to 10^6^ cells per ml. siRNA (IDT), at a concentration of either 12.5 or 6.25 nM, was diluted in Opti-MEM and combined with Dharmafect 1 Transfection Agent (NC1308404; Horizon Discovery) at a final concentration of 0.2% vol/vol. The concentration was predetermined by titrating down siRNA concentrations with an initial qPCR for each siRNA sequence for an ∼50% reduction in the target RP gene. Transfection Opti-MEM mix was incubated at room temperature for 20 min before addition to the cell suspension in Opti-MEM. The cells were then incubated at 37°C with 5% CO_2_ for 4 h. After this incubation period, the transfection medium was removed and replaced with RPMI supplemented with FBS and glucose. The cells were harvested 48 h post-knockdown procedure with details provided in the next section.

After the knockdown procedure, cells were centrifuged at 600 × *g* for 5 min and washed with chilled PBS (21-040-CM; Corning). An aliquot was flash-frozen in liquid nitrogen for ADP/ATP assay analysis, and the cells were then subjected to a second centrifugation under the same conditions. The supernatant was discarded, and 350 μl of Trizol was added to the cell pellet. The cells were briefly vortexed before undergoing standard phenol–chloroform precipitation. Next, the cell lysate was treated with Turbo DNase (AM2238; Thermo Fisher Scientific) following the manufacturers guidelines and then subjected to acidic phenol-chloroform extraction (AM9720; Thermo Fisher Scientific) for RNA purification. The purified RNA was then converted into cDNA using Superscript III Reverse Transcriptase (18080-093; Thermo Fisher Scientific). Quantitative PCR (qPCR) was performed using the PowerUP SYBR Green Master Mix Solution (A25779; Thermo Fisher Scientific) following the manufacturers protocol. The qPCR reactions were performed on a Fast 96-well plate (4346907; Thermo Fisher Scientific) to quantify gene expression levels and knockdown efficiency.

Measurement of relative expression was performed using comparative methods through ΔΔCt measurement. Ct (Cycle threshold) values were obtained from qPCR amplification of samples with either their targeted primer or housekeeping primer (GAPDH) in three technical replicates and averaged. The ΔCt value was calculated by subtracting the average Ct value of a targeted primer from the average Ct value of a housekeeping primer. The ΔΔCt was calculated by subtracting the ΔCt of the treatment group with the ΔCt of the control group. The relative expression level was computed with the following equation: Expression level = 2^(−ΔΔCt) (Livak and Schmittgen, 2001).

siRNA sequences used in this study are provided below and in Table S9.

### K562 ADP/ATP assay

Following the collection of cells in PBS as described, the ADP/ATP ratio was determined using the ADP/ATP Assay Kit (MAK135-1KT; Sigma-Aldrich). The assay was conducted on a Glomax Luminometer (Promega), following the guidelines provided by the manufacturer.

### K562 mitotracker accumulation assay

In the assay for mitochondrial membrane staining, K562 cells were quantitatively stained using 50 nM Mitotracker CMXRos (M7512; Thermo Fisher Scientific) alongside 2 µM Hoechst (62249; Thermo Fisher Scientific) for nuclear staining for a duration of 30 min. After staining, the cells were washed in PBS and centrifuged at 600 × *g* for 5 min. Subsequently, the cells were resuspended in LiveCell Imaging Solution (A14291DJ; Thermo Fisher Scientific). Fluorescent imaging was performed using a 40× (NA 1.3) objective on a Leica SPE microscope with a Leica K5 microscope camera. Images were taken using Leica Application Suite X, and the intensity of MitoTracker staining per area within the cells was quantitatively analyzed using ImageJ software.

### Online supplemental material

Fig. S1 illustrates that RP haploinsufficiency causes developmental delays and delayed brood size in *C. elegans* without impacting lifespan and shows that overall protein translation remains largely unaffected in these mutants. Fig. S2 investigates stress responses, revealing that RP mutants exhibit altered survival rates under oxidative stress. Stress response pathways, involving skn-1 and daf-16, remain comparable with wild-type without any RP haploinsufficiency. Fig. S3 illustrates gene expression and TE differences, with significant changes in RP genes and confirming that ribosomal RNA ratios are unaltered in RP haploinsufficient animals. Fig. S4 assesses mitochondrial ETC components expression at the protein level, as well as mitochondrial ribosome expression at the RNA, TE, and protein levels, it also includes quality controls for mitochondrial staining, and mitochondrial DNA coverage plots, concluding that mitochondrial abundance is largely maintained despite RP haploinsufficiency. Fig. S5 identifies enriched GO categories related to translational control shared between *C. elegans* and humans and evaluates the effects of RP copy loss and gene knockdown on gene expression at the RNA and translation level. Tables S1, S2, S3, S4, S5, S6, S7, S8, and S9 provide detailed datasets supporting these findings, including proteomic and RNA-seq analyses, GO enrichments, and correlations between ribosomal and mitochondrial gene expression. Data S1 provides data underlying Fig. 1 E. Data S2 provides data underlying Fig. 2 C. Data S3 provides more data underlying Fig. 2 C. Data S4 provides data underlying Fig. 5 A. Data S5 provides data underlying Fig. 5 B. Data S6 provides data underlying Fig. 5 C. Data S7 provides data underlying Fig. 5 E. Data S8 provides data underlying Fig. 5 F. Data S9 provides data underlying Fig. 5 G. Data S10 provides data underlying Fig. 5 H. Data S11 also provides data underlying Fig. 5 H. Data S12 provides more data underlying Fig. 5 H. Data S13 provides data underlying Fig. 7 B. Data S14 provides data underlying Fig. 7 C. Data S15 provides data underlying Fig. S1 C. Data S16 provides data underlying Fig. S1 D. Data S17 provides more data underlying Fig. 1 D. Data S18 provides more data underlying Fig. S2 A. Data S19 provides data underlying Fig. S2 C. Data S20 provides data underlying Fig. S2 E.

## Supplementary Material

Table S1 shows proteomics analysis of all RP haploinsufficient mutants, including predictions for fold changes and P values calculated using DEP.

Table S2 shows RNA-seq analysis of all RP haploinsufficient mutants, including predictions for fold changes and P values calculated using EdgeR.

Table S3 shows significantly enriched gene ontology categories for genes that were significantly over- or under-expressed i at the RNA level in all RP haploinsufficient mutants are shown (P_adj_ < 0.05).

Table S4 shows RNA, Ribo-Seq, and Translation Efficiency (TE) fold changes and P values for all RP haploinsufficient mutant animals, analyzed using EdgeR.

Table S5 shows significantly enriched gene ontology (GO) categories for genes that were significantly over- or under-expressed at the RNA and TE level, either unidirectionally or bidirectionally, in all RP haploinsufficient mutants (P_adj_ < 0.05).

Table S6 shows RNA, Ribo-Seq, and Translation Efficiency (TE) fold changes and P values in response to *shRPL5* and *shRPS19* knockdown in hematopoietic cells, analyzed using EdgeR.

Table S7 shows RNA, Ribo-Seq, and Translation Efficiency (TE) fold changes of all orthologous genes between *C. elegans* and humans in response to *RPL5* reduction, analyzed using EdgeR.

Table S8 shows all Ribo-Mito interactions with a rho score higher than 0.75 at the RNA and TE level are listed.

Table S9 shows strains generated or utilized in this study, as well as DNA and RNA sequences used.

Data S1 provides data underlying Fig. 1 E.

Data S2 provides data underlying Fig. 2 C.

Data S3 provides more data underlying Fig. 2 C.

Data S4 provides data underlying Fig. 5 A.

Data S5 provides data underlying Fig. 5 B.

Data S6 provides data underlying Fig. 5 C.

Data S7 provides data underlying Fig. 5 E.

Data S8 provides data underlying Fig. 5 F.

Data S9 provides data underlying Fig. 5 G.

Data S10 provides data underlying Fig. 5 H.

Data S11 also provides data underlying Fig. 5 H.

Data S12 provides more data underlying Fig. 5 H.

Data S13 provides data underlying Fig. 7 B.

Data S14 provides data underlying Fig. 7 C.

Data S15 provides data underlying Fig. S1 C.

Data S16 provides data underlying Fig. S1 D.

Data S17 provides more data underlying Fig. 1 D.

Data S18 provides data underlying Fig. S2 A.

Data S19 provides data underlying Fig. S2 C.

Data S20 provides data underlying Fig. S2 E.

SourceData FS1 is the source file for Fig. S1 D.

SourceData FS4 is the source file for Fig. S4 C.

## Data Availability

Data points or raw data images are all provided in the Texas Data Repository, University of Texas at Austin Dataverse Collection under Sarinay Cenik Lab Dataverse. The link is as follows: https://dataverse.tdl.org/dataset.xhtml?persistentId=doi:10.18738/T8/AVZD3L. All high-throughput datasets were uploaded to NCBI GEO (GSE280071). DNA oligo sequences, siRNA sequences, and plasmids used in this study are available in Table S9.
