# Supplementary figures and images for: Differential impacts of ribosomal protein haploinsufficiency on mitochondrial function
Source: J Cell Biol. 2025 Jan 9;224(3):e202404084. doi: 10.1083/jcb.202404084 (PMC11716151; doi:10.1083/jcb.202404084)

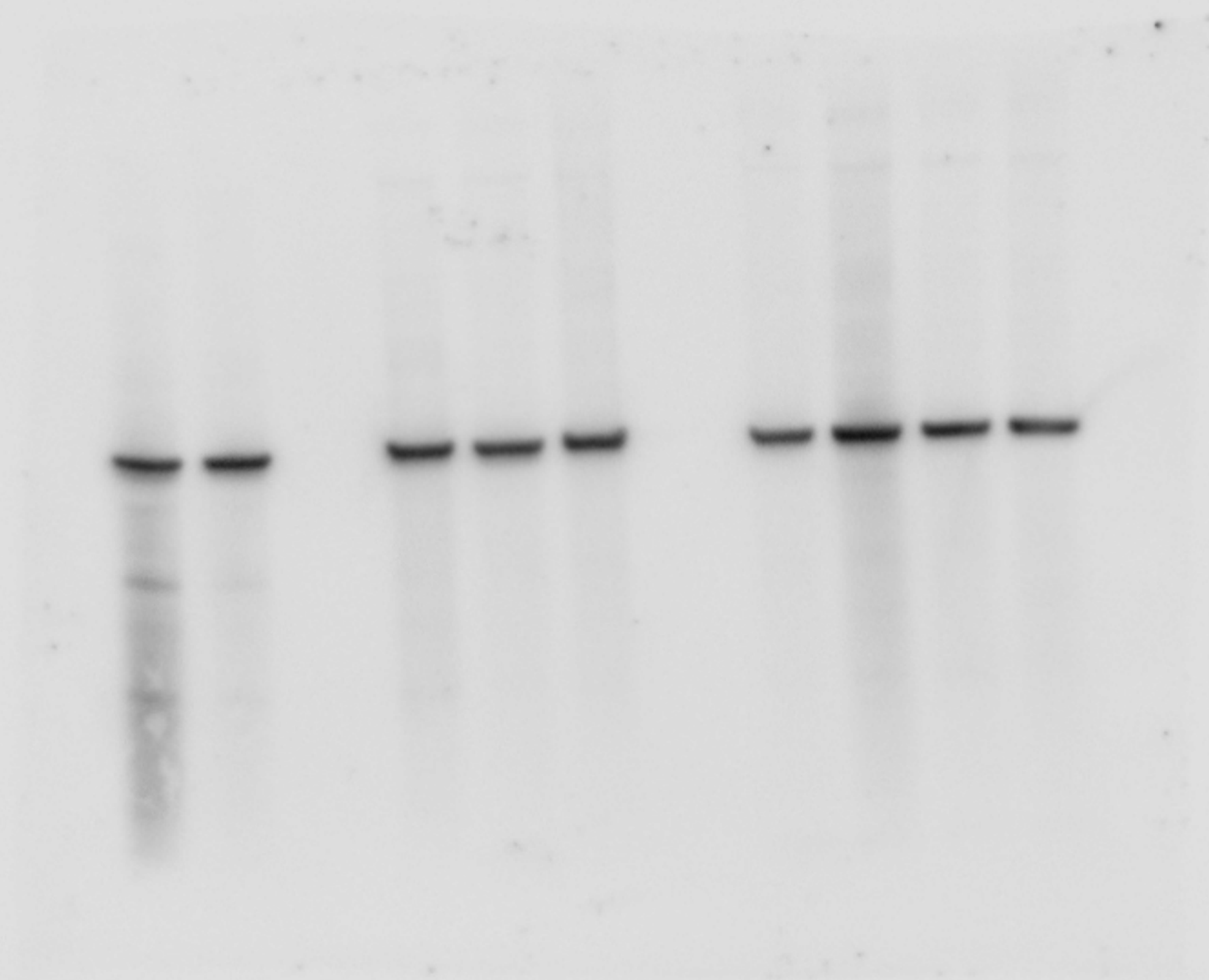

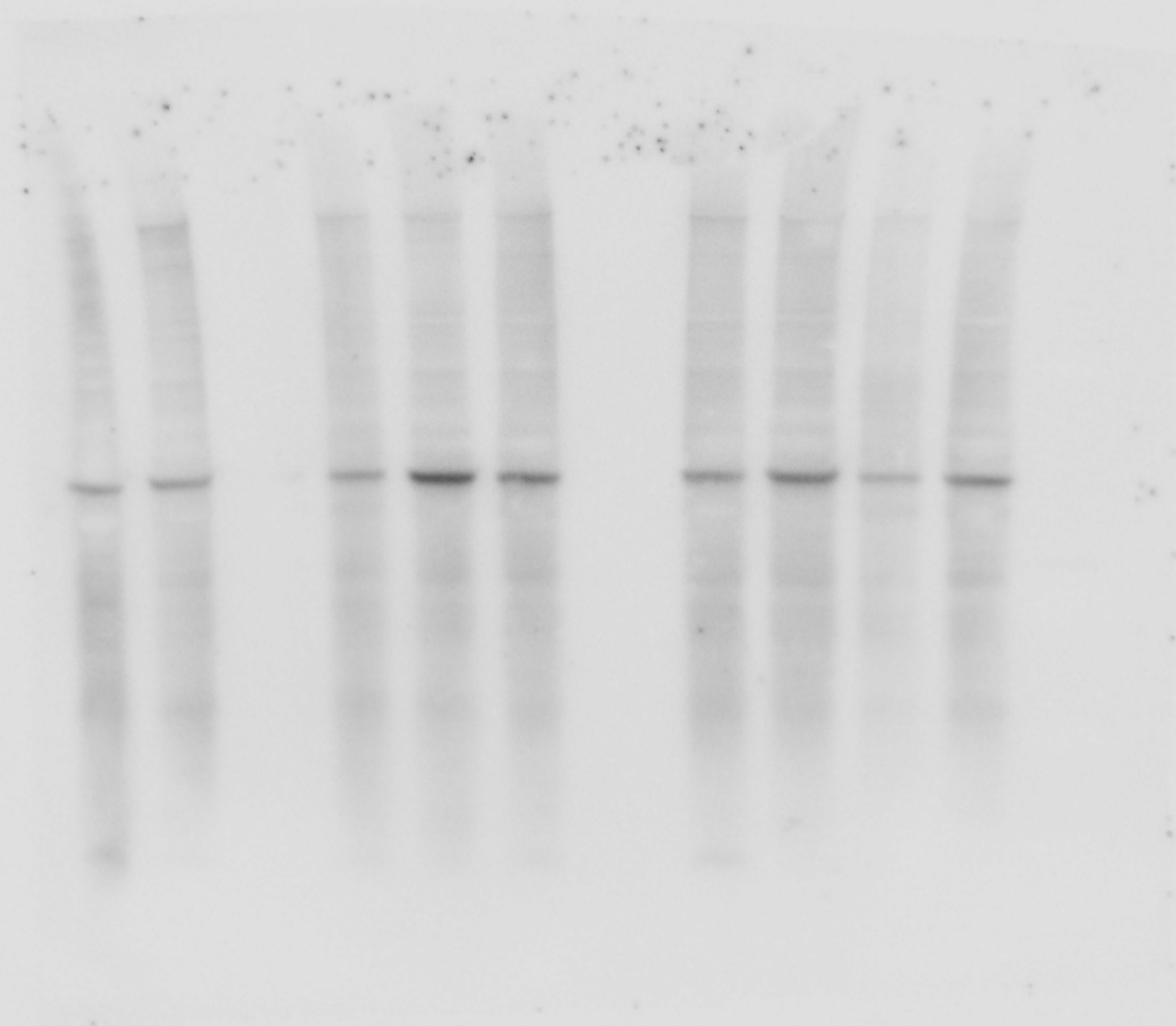

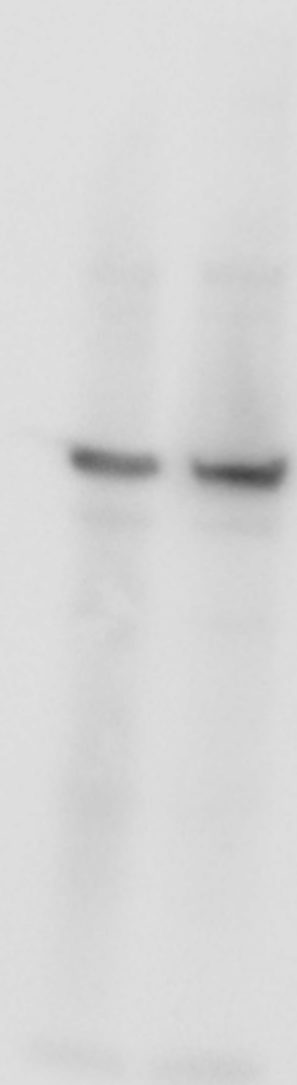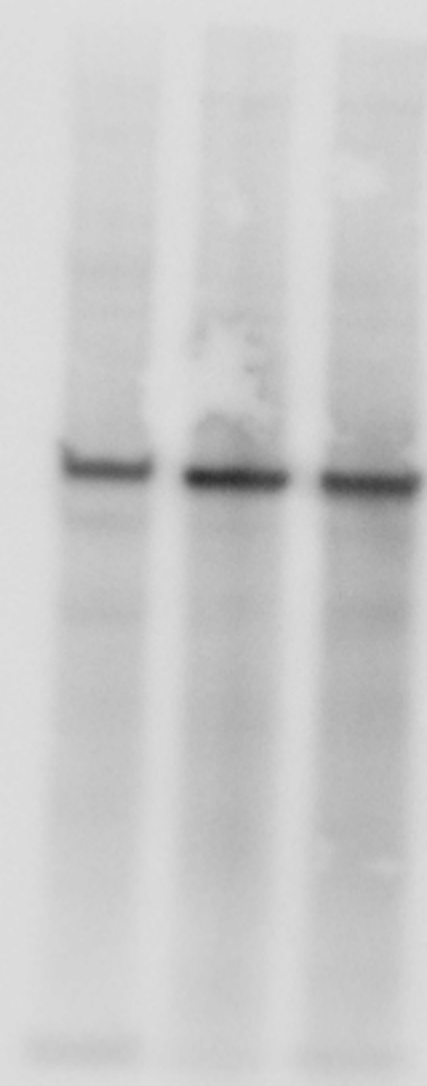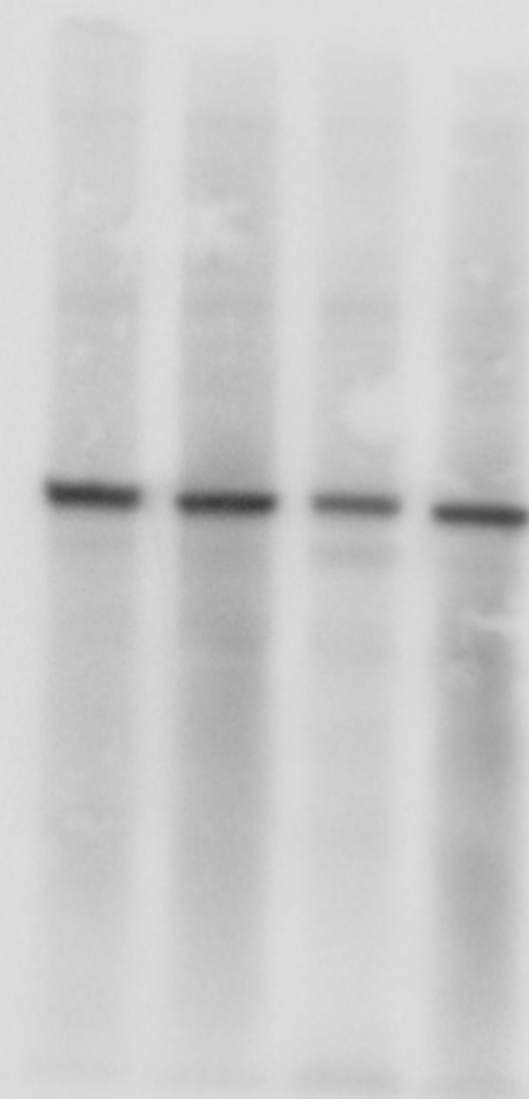

1 1 1 1

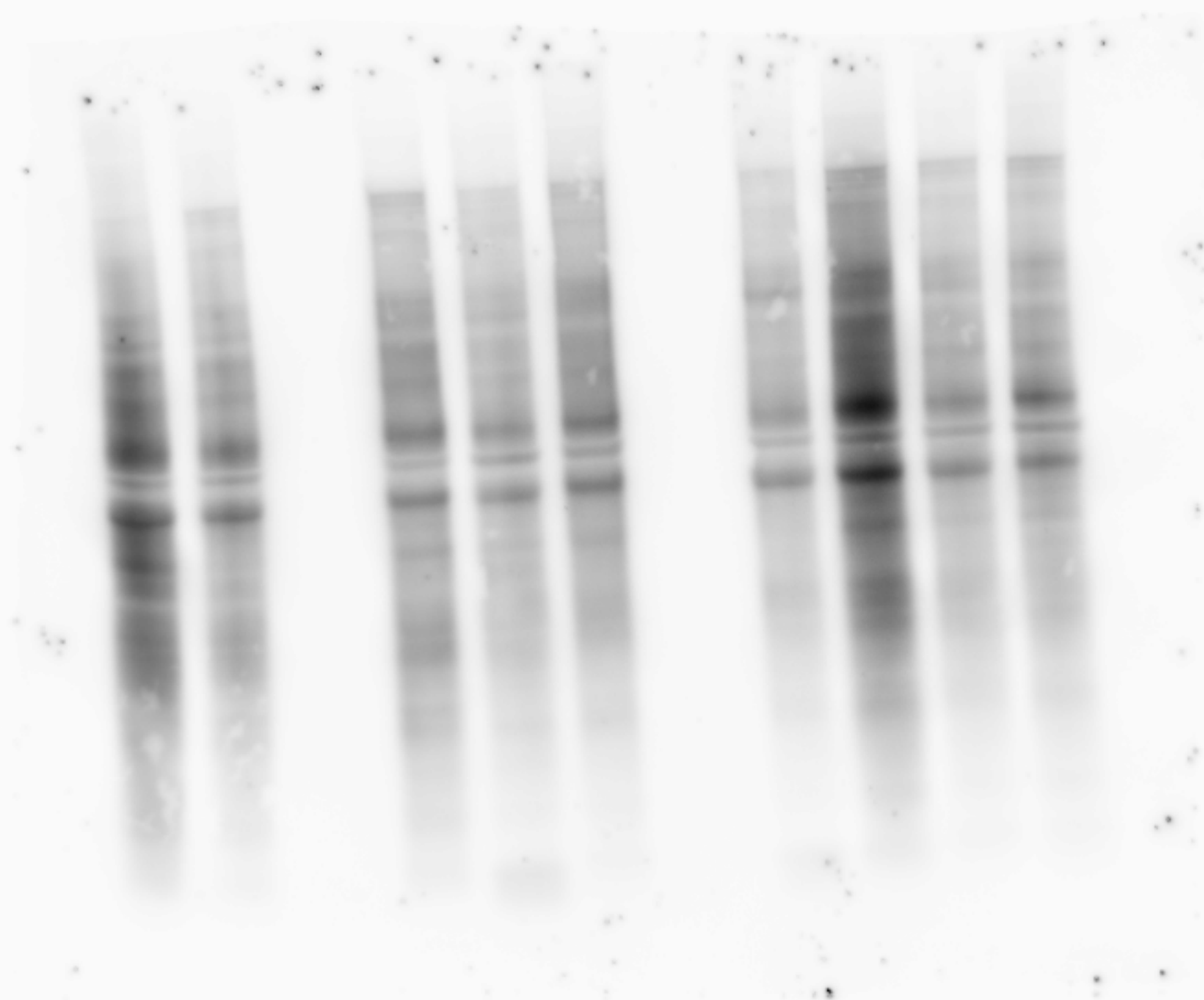

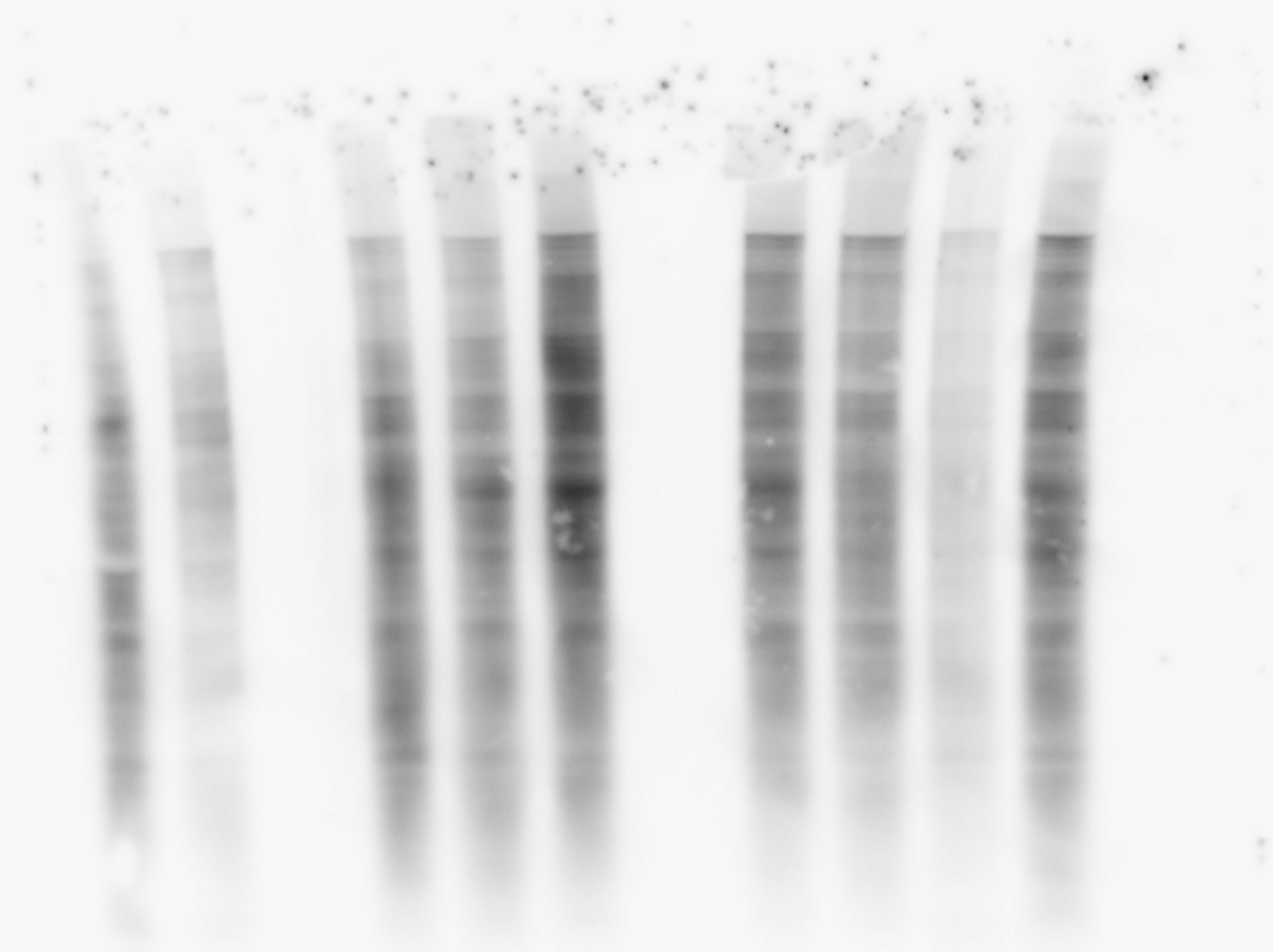

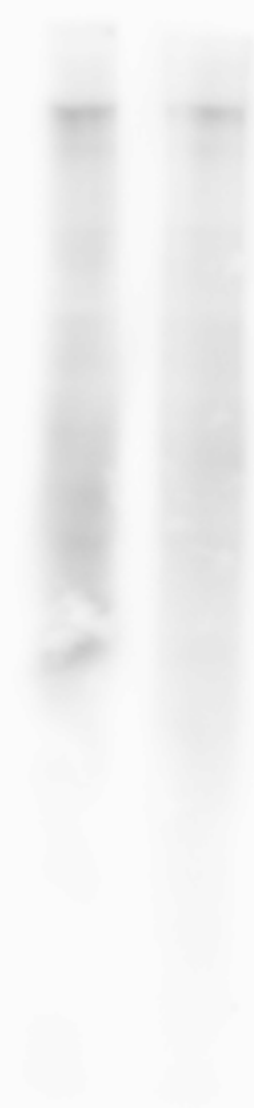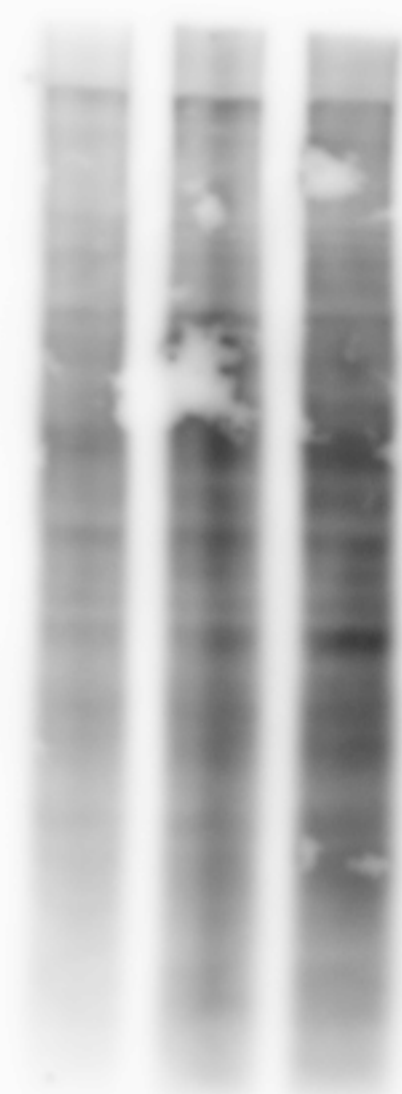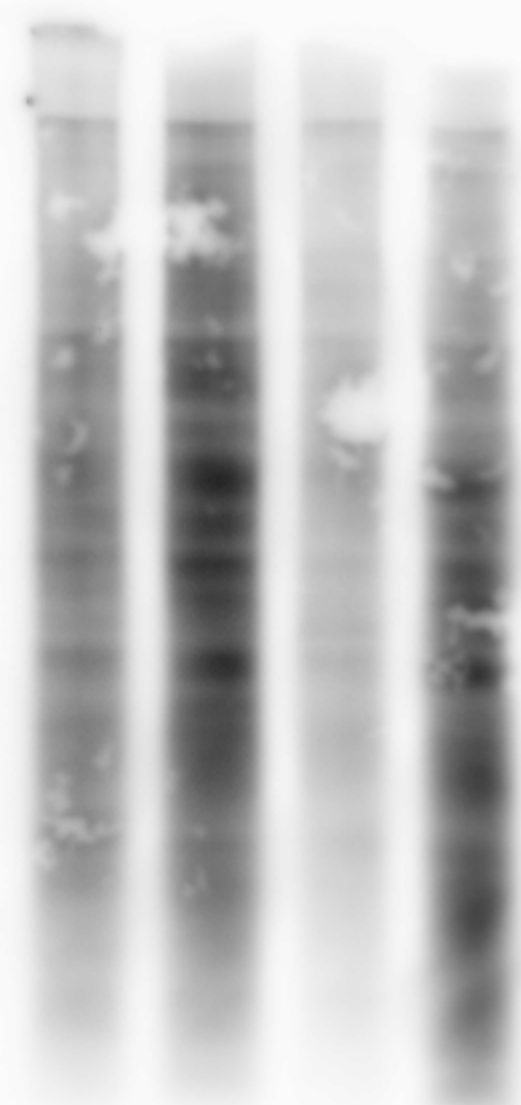

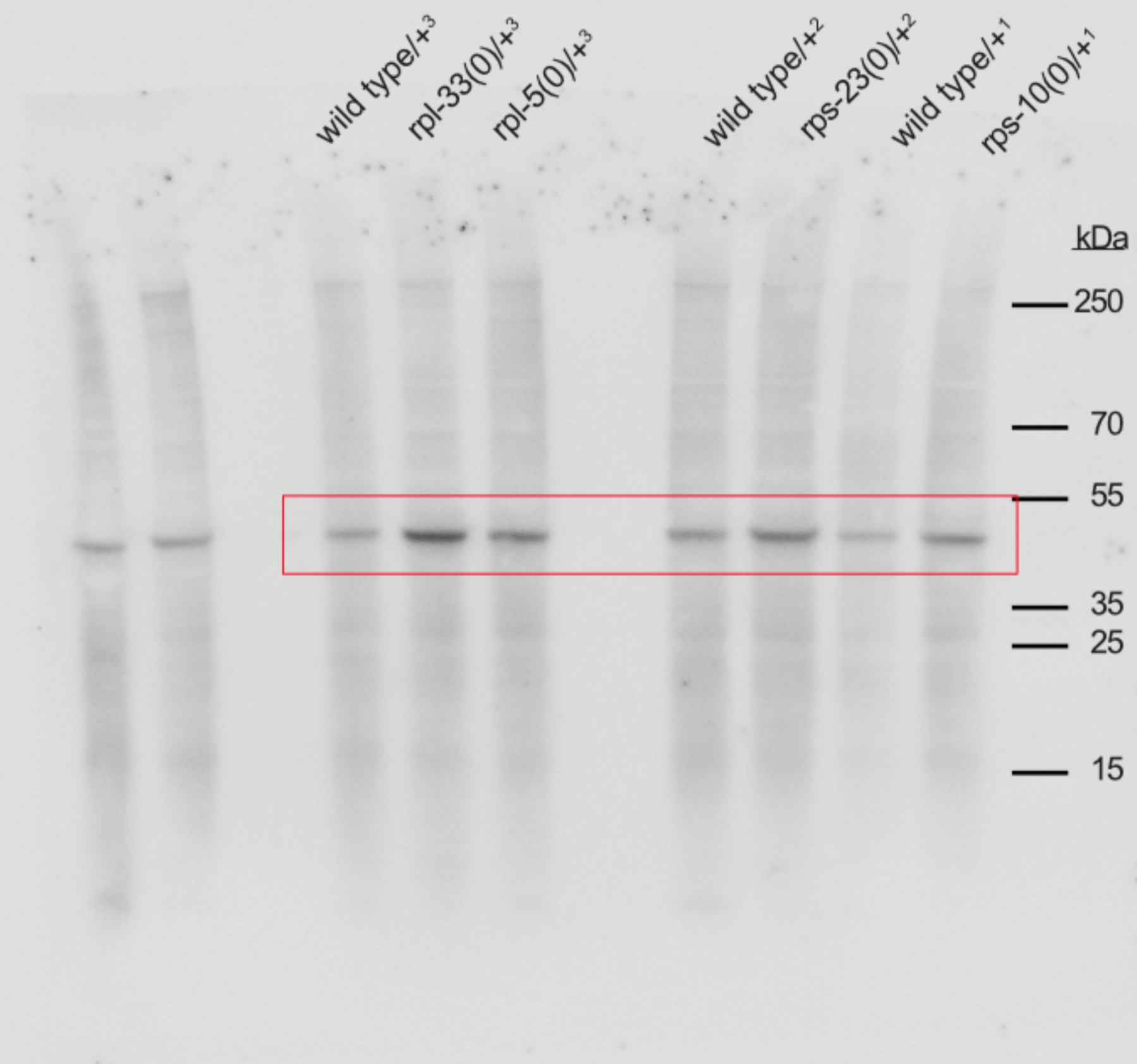

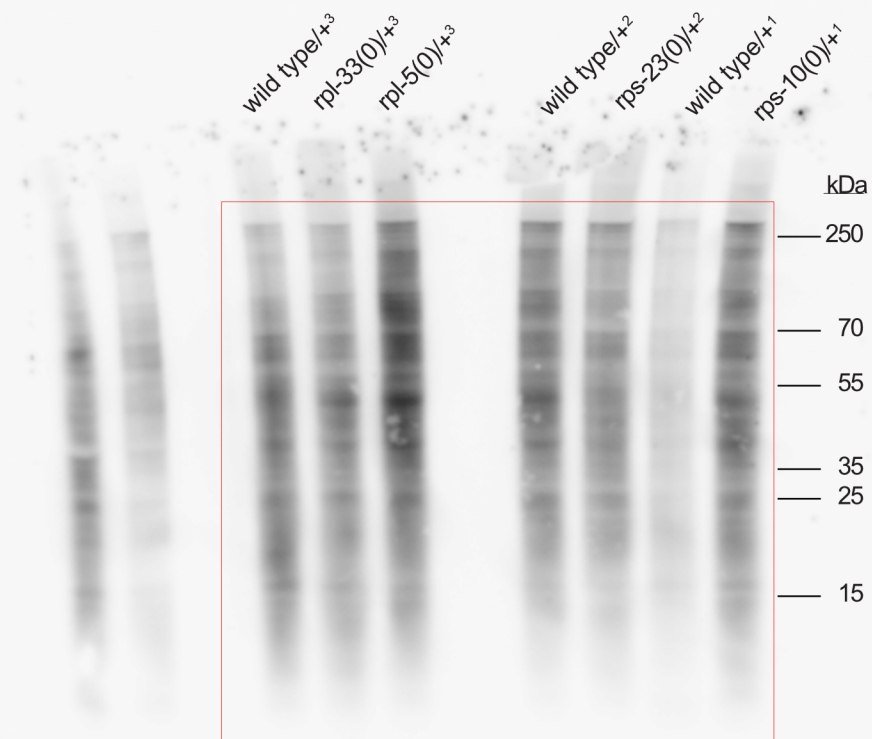

Supplement: SourceData FS1 — is the source file for Fig. S1 D. [file jcb_202404084_sourcedatafs1.pdf]

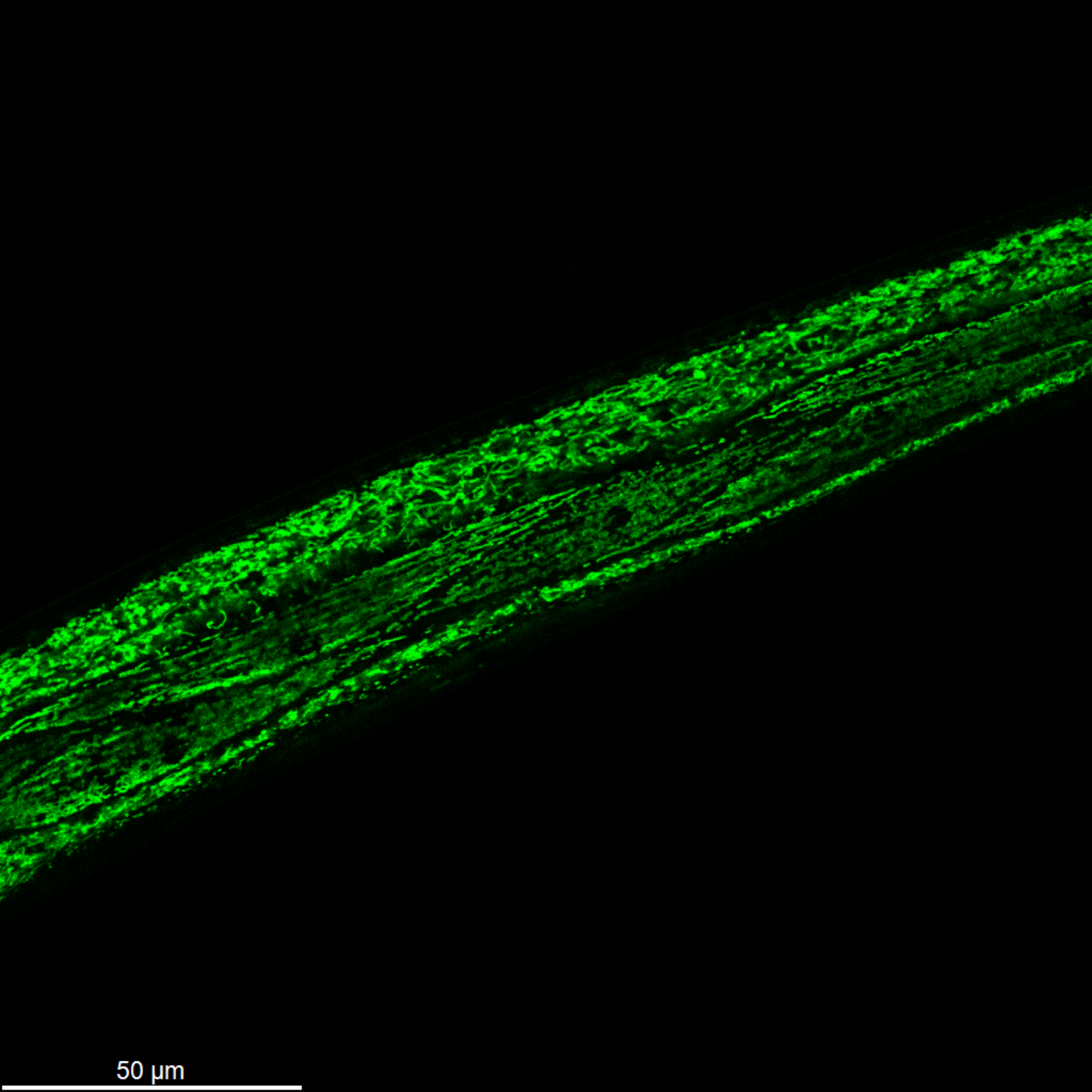

50  $\mu\text{m}$

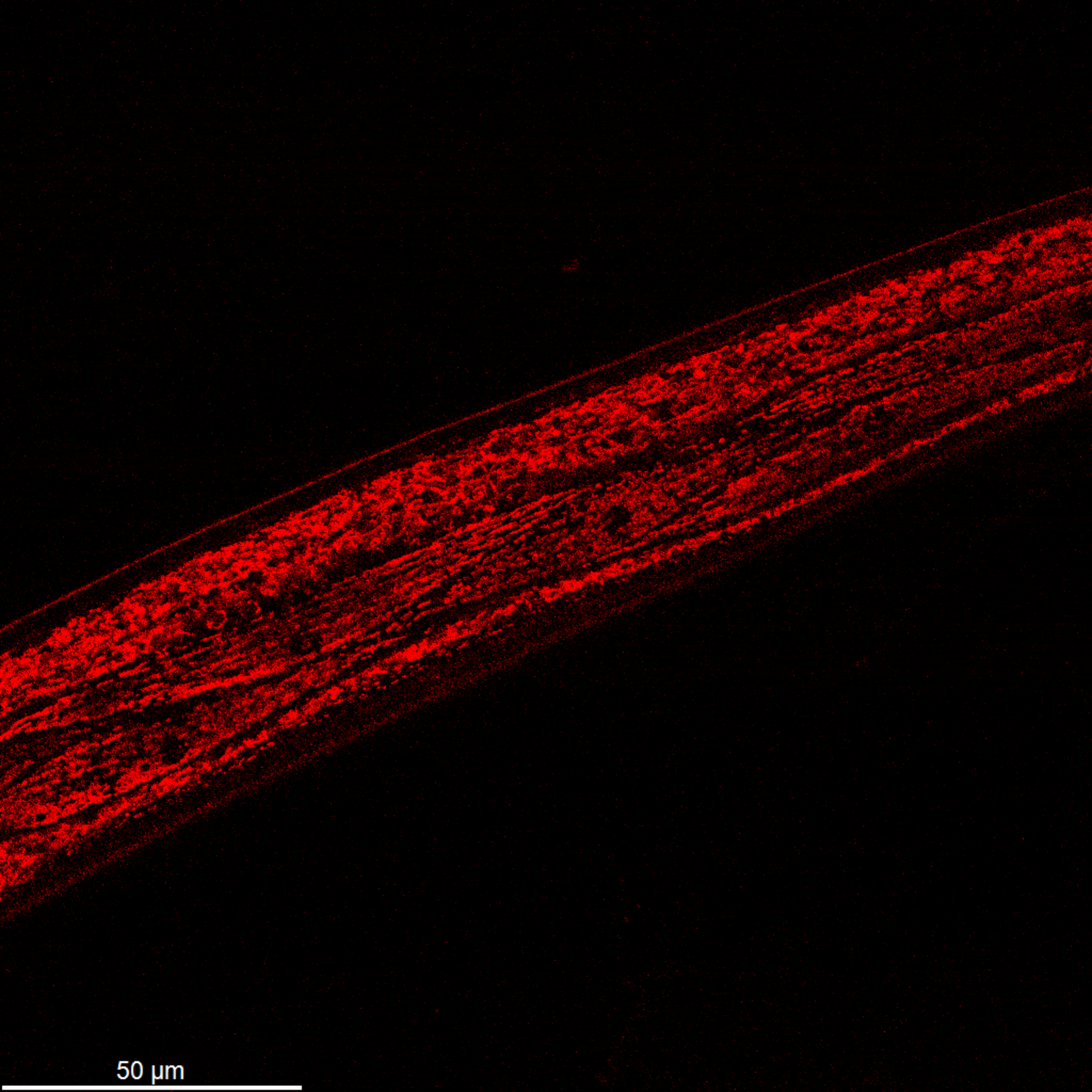

50 μm

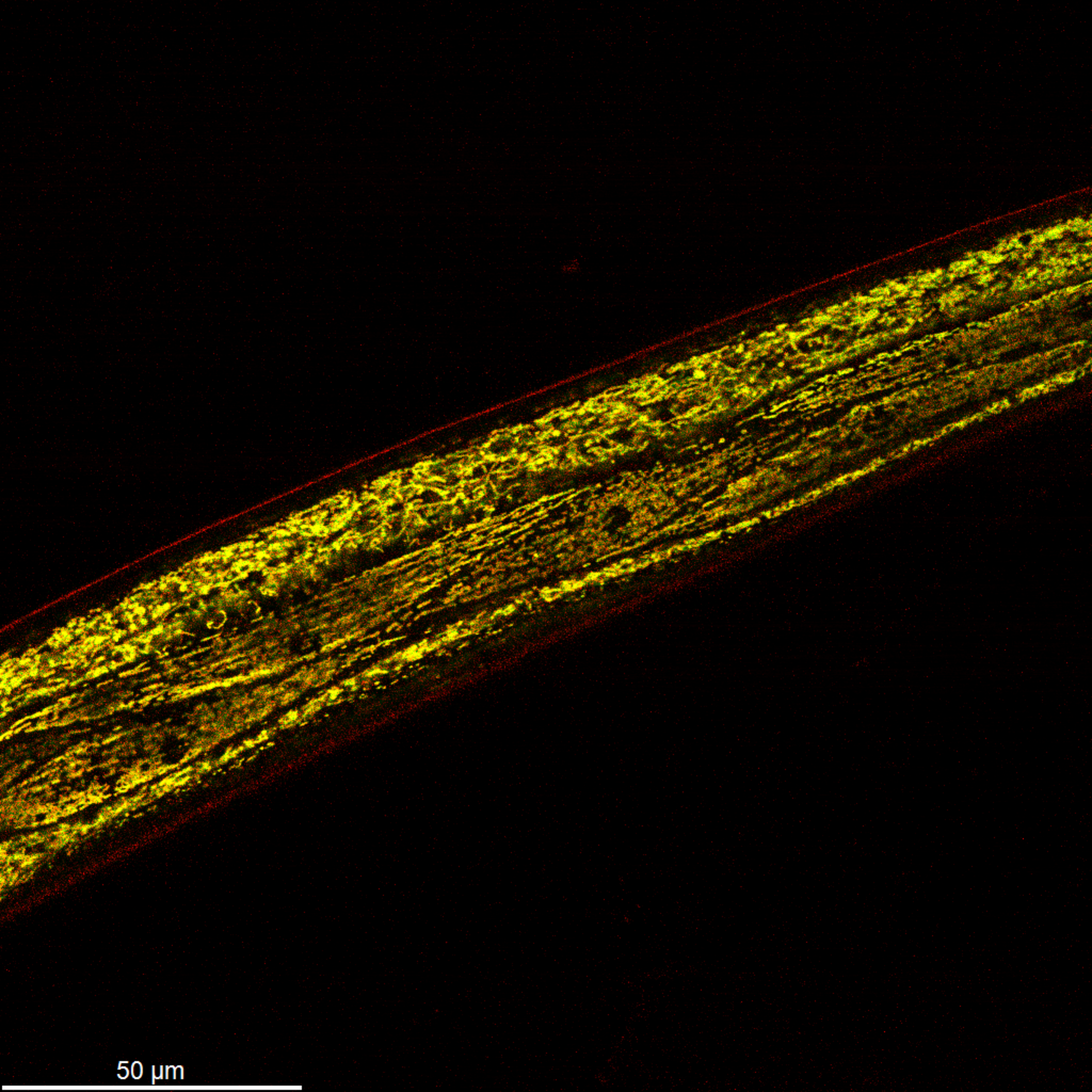

50  $\mu\text{m}$

Supplement: SourceData FS4 — is the source file for Fig. S4 C. [file jcb_202404084_sourcedatafs4.pdf]
